# Homeodomain Involvement in Nuclear HOX Protein Homo- and Heterodimerization

**DOI:** 10.3390/ijms26010423

**Published:** 2025-01-06

**Authors:** Damien Marchese, Laetitia Evrard, Isabelle Bergiers, Ludovic Boas, Justine Duphénieux, Maryse Hermant, Tamara Pringels, Fisnik Zeqiri, Marc Pirson, Jean-Claude Twizere, Françoise Gofflot, René Rezsohazy, Laure Bridoux

**Affiliations:** 1 Louvain Institute of Molecular Science and Technology, Université catholique de Louvain, 5 (L7.07.10) Place Croix du Sud, 1348 Louvain-la-Neuve, Belgium; 2 Gembloux Agro-Bio Tech, University of Liège, Avenue de la Faculté de Gembloux, 5030 Gembloux, Belgium

**Keywords:** transcription factor, HOX, HOXA1, TALE, dimerization, nuclear localization, nucleus, homeodomain, development

## Abstract

*HOX* genes play essential roles in patterning the anteroposterior axis of animal embryos and in the formation of various organs. In mammals, there are 39 *HOX* genes organized into four clusters (HOXA–D) located on different chromosomes. In relationship with their orderly arrangement along the chromosomes, these genes show nested expression patterns which imply that embryonic territories co-express multiple *HOX* genes along the main body axis. Interactomic database entries, as well as a handful of publications, support that some HOX proteins can form homodimers or interact with other HOX proteins. However, the consequences of HOX protein interactions have been poorly investigated and remain largely elusive. In this study, we compiled a repository of all HOX–HOX interactions from available databases, and taking HOXA1, HOXA2, and HOXA5 as examples, we investigated the capacity of HOX proteins to form homo- and heterodimers. We revealed that while the DNA-binding domain, the homeodomain, is not necessary for HOXA1 homodimerization, the nuclear localization of the dimerization is dependent on the homeodomain, particularly the integrity of the third helix of HOXA1. Furthermore, we demonstrated that HOXA1 can influence the localization of HOXA1 when it is deprived of the homeodomain, increasing its abundance in the chromatin-containing fraction. Moreover, HOXA1 nuclear homodimerization occurs independently of the integrity of the hexapeptide and, consequently, of its well-known interactor, the homeodomain protein PBX. These results hint at a potential involvement of dimerization in the complex landscape of HOX regulatory mechanisms.

## 1. Introduction

Gene regulation is at the heart of most biological processes. Deciphering regulatory mechanisms, from transcription initiation to protein decay, is therefore crucial to the understanding of life. The transcription factors (TFs) of the HOX protein family are critical to gene regulation processes involved in animal embryogenesis. Misexpression of *HOX* genes is associated with dramatic alterations in body plan, limb formation, or cell differentiation [1,2]. Although the functions of HOX in the embryo and in other biological contexts have been quite well-characterized, two major challenges towards a full understanding of HOX functions remain: (i) deciphering the molecular mechanism underlying the genetic interactions of *HOX* and (ii) understanding how HOX proteins are regulated at a cellular level to achieve their functional specificity.

In mammals, *HOX* genes are organized in four genomic clusters (HOXA–D) located on different chromosomes and classified into 13 paralog groups (PGs) subdivided as anterior (PG1–4), central (PG5–8), and posterior (PG9–13) PGs [1,3]. *HOX* gene expression is collinear in space and time, with their rostral limit of expression being defined by their position along the clusters (i.e., genes located at the 3′ end of the complexes are expressed earlier and show a more rostral limit of expression along the main body axis than genes located closer to the 5′ end). This gives rise to embryo territories expressing distinct combinations of *HOX* genes along the body axis, with posterior domains expressing more *HOX* genes than anterior ones [4].

HOX proteins, and more globally, TFs containing homeodomains (HDs), exhibit high conservation in amino acid residues that come into contact with DNA (previously reviewed [1]). Consequently, it is unsurprising that biochemical analyses revealed a tendency of HOX proteins to bind highly similar DNA sequences in vitro, such as AT-rich sequences. However, in sharp contrast to their in vitro behavior, they have been reported to regulate distinct developmental pathways in vivo. The mechanism underlying how HD proteins—and HOX in particular—may achieve DNA binding specificity and regulate distinct targets, despite the high HD sequence similarity, remains vastly unknown. Several non-exclusive regulatory mechanisms could partially explain this paradoxical phenomenon (reviewed by [5,6]). For instance, HOX proteins have been shown to interact with TALE (three-amino acid loop extension) homeodomain proteins (MEIS, PREP, and PBX). Although these interactors modulate the DNA binding selectivity of HOX proteins, TALE HD proteins interact with most HOX proteins and are therefore unlikely to be the sole actors defining HOX functional specificity (previously reviewed [7,8]). Interactions with more specific partners could modify the behavior of HOX in a context-dependent manner. In this regard, large-scale interaction screenings revealed the ability of HOX proteins to bind a large repertoire of proteins involved in a range of different biological processes [9,10,11,12,13].

A handful of studies reported that HOX proteins can form homodimers and heterodimers with other HOX proteins [9,13,14,15,16,17]. The formation of such complexes could be a key piece in the puzzles that are the HOX specificity paradox and the genetic interactions displayed between *HOX* genes. Indeed, dimer formation could modulate various aspects of the proteins’ properties, including their stability, intracellular localization, DNA binding, or transcriptional activity, in turn potentially contributing to their specific functions. However, to date, the molecular modalities underlying HOX–HOX protein interactions, as well the concrete impact of these interactions, both at cellular and organismic levels, remain largely unexplored.

Cooperative DNA binding, where the binding of a second protein is facilitated or depends on the binding of the first, has been predicted in silico for certain HD-containing dimers and experimentally confirmed in some instances [18,19,20]. Specifically, for HOX proteins, four combinations (SCR–SCR, UBX–UBX, HOXB2–HOXB13, and HOXD12-HOXA3) were reported to cooperatively bind DNA [16,19,21,22,23]. However, it remains unclear in most cases whether these cooperativities involve direct protein–protein contact.

Interestingly, in *Drosophila*, Sex Combs Reduced (SCR), the homolog of mammalian HOX5 paralogs, was shown to homodimerize [16,17]. Furthermore, the glutamic acid located at position 19 in the HD of SCR was identified as important for the formation of this complex, while this residue was shown not to be required for DNA binding of the protein [16]. Further analysis reported that SCR dimers exhibit a widespread association with DNA and show a slow diffusion rate compared to monomeric SCR [16,17]. In mammals, a physical interaction between HOXA2 and HOXA3 was detected in cell culture. However, though these proteins share common binding sites in the developing embryo, an interaction was not observed at the DNA level in in vitro conditions [15,24]. HOXA1 homodimerization and its possible functional consequences were also the focus of several publications [9,25,26]. An isoform of HOXA1(1-399) lacking the HD and containing a unique carboxy terminus was shown to be transcriptionally inactive while retaining its ability to interact with the full-length HOXA1 [26]. Moreover, BiFC experiments confirmed the presence of HOXA1 homodimers in the nucleus [9].

Put together, these data support the possibility that HOX protein homodimerization and heterodimerization might be a shared property throughout the HOX family. In the present study, starting with data mining and the testing of distinct combinations of HOX protein interactions, we conducted an in-depth investigation into the molecular mechanisms underlying HOX dimerization, focusing on different parameters such as the cellular localization of the dimer, the region required for the interaction, and the involvement of cofactors.

## 2. Results

### 2.1. Interactions Between HOX Proteins Are a Widely Shared Property of the HOX Protein Family and Mainly Occur in the Nucleus

**Figure 1:**
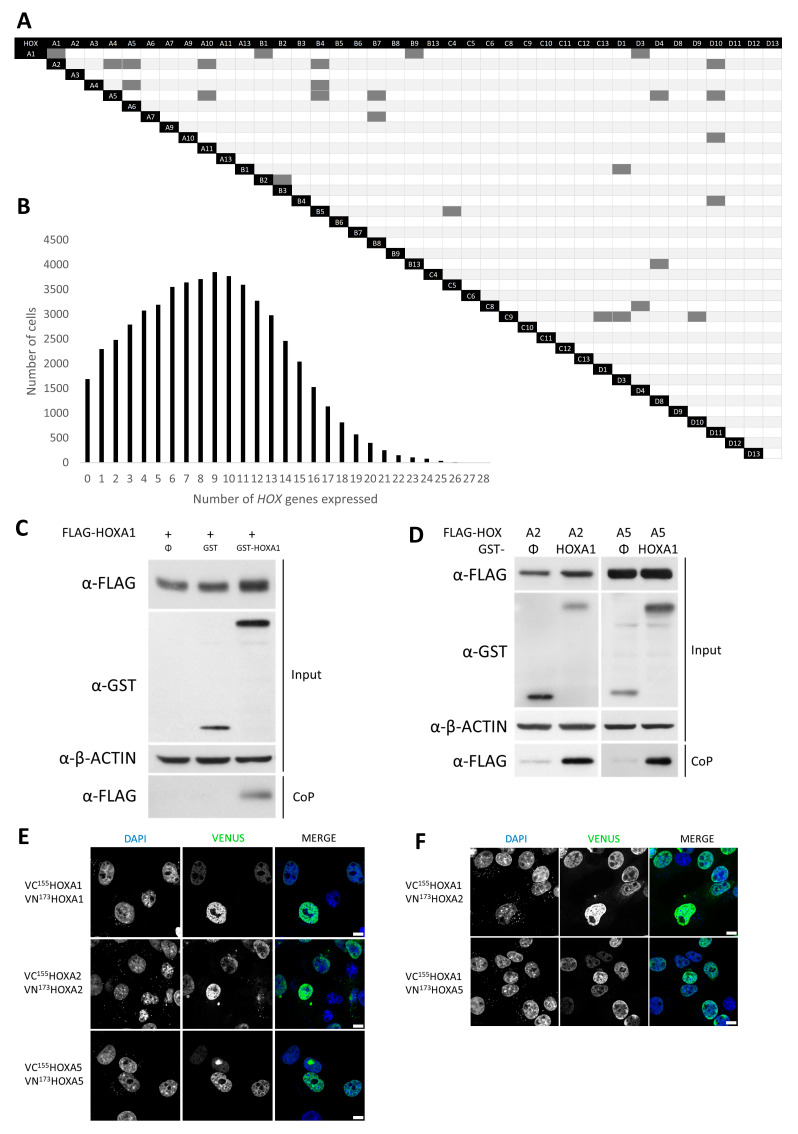
Interactions between HOX proteins are widely shared. (**A**). **Reported HOX interactions**. Compilation of HOX–HOX interactions (human and murine) reported in the BIOGRID database. The dark grey boxes in the double-entry table indicate reported interactions. (**B**). ***HOX* genes are co-expressed.** Visualization of the number of cells co-expressing distinct *HOX* genes in the developing spinal cord. Single-cell sequencing data were extracted from Delile et al., 2019 [27], using normalized counts. (**C**,**D**). **HOXA1 forms homo- and heterodimers**. Coprecipitation (CoP) assay. HEK293T cells were transfected with a combination of plasmids coding for FLAG–HOXA1, FLAG–HOXA2, or FLAG–HOXA5, and GST or GST–HOXA1. Proteins were analyzed by Western blotting before (Input) and after coprecipitation on glutathione beads (CoP). Antibodies were directed against GST or FLAG tags. β-ACTIN detection was used as the loading control. Presented blots are representative of at least three independent experiments (N ≥ 3). (**E**,**F**). **HOX dimers localize in the nucleus**. Bimolecular Fluorescence Complementation (BiFC). COS-7 cells were transfected with plasmids coding for VC^155^HOXA1 and VN^173^HOXA1, VC^155^HOXA2 and VN^173^HOXA2, VC^155^HOXA5 and VN^173^HOXA5, VC^155^HOXA1 and VN^173^HOXA2, or VC^155^HOXA1 and VN^173^HOXA5. Upon interaction between the partner proteins, the VN^173^ and VC^155^ moieties of the Venus fluorescent protein are brought together and generate a green fluorescent signal. Nuclei were stained with DAPI (blue). Pictures were obtained using confocal microscopy. Scale bars = 10 μm. Presented pictures are representative of at least three independent experiments (N ≥ 3).

As introduced above, HOX–HOX dimerizations were reported for several HOX proteins. To assess whether this type of interaction was a shared feature of the HOX family, we compiled all HOX–HOX interactions reported in the BioGRID database and highlighted 26 possible interactions in mice and humans, mainly revealed from large interactomic screenings (Figure 1A, Supplemental Table S1). Additionally, seventeen genetic and nine protein interactions between HOX proteins have also been reported in Drosophila (Supplemental Figure S1A,B, Supplemental Table S2).

Research over the last decades has clearly established that *HOX* genes are expressed in overlapping territories in developing embryos, resulting from their collinear expression [4]. However, whether several *HOX* genes are co-expressed within the individual cells of these overlapping territories remains to be investigated. Data regarding co-expression of *HOX* genes at a cellular level would be highly relevant to understanding the functional impact of the identified HOX heterodimerization. Single-cell analysis of developing mouse spinal cords, where the expression of 35 out of 39 *HOX* genes was detected (Supplemental Figure S1C), reported *HOX* co-expression in 93% of the cells, with only a minority of cells expressing ≤1 *HOX* gene (no *HOX* = 3%, one *HOX* = 4% of the cells) (Figure 1B) [27]. In particular, *HOXA1*, used as a model for the present study, was found to be expressed in combination with other *HOX* genes in 99.6% of the cells (Supplemental Figure S1D).

In order to validate HOX homo- and heterodimerization detection in a cellular model and further characterize the molecular modality of such interactions, we focused on HOXA1 and HOXA2, two anteriorly expressed proteins, and HOXA5, which belongs to a more posteriorly expressed paralog group. Coprecipitation of proteins was performed using transiently transfected HEK293T cells. Proteins were expressed fused either with a FLAG tag or a glutathione S-transferase (GST) tag; the GST tag further allows the precipitation of the proteins thanks to glutathione beads. In the presence of GST–HOXA1, a band corresponding to FLAG–HOXA1 was detected by Western blotting, suggesting that the two proteins can form a homodimer in HEK293T cells (Figure 1C). Similarly, HOX heterodimerizations between HOXA1 and HOXA2, as well as HOXA1 and HOXA5, were further confirmed by coprecipitation (Figure 1D).

Further characterization of the interaction was performed via bimolecular fluorescence complementation (BiFC) to determine the cellular localization of HOX dimers. To this end, HOXA1, HOXA2, and HOXA5 were fused to the N-terminal (VN^173^) and the C-terminal (VC^155^) moieties of the Venus fluorescent protein. Using the complementation of the Venus protein, we showed that the signals emitted by HOXA1–HOXA1, HOXA2–HOXA2, and HOXA5–HOXA5 complexes were invariably stronger than BiFC controls (namely VN^173^HOX-VC^155^, VN^173^-VC^155^HOX, and VN^173^-VC^155^, Supplemental Figure S1E) and were mainly located in the nucleus (Figure 1E). Similarly to the homodimerization results (Figure 1E), the BiFC signal associated with HOXA1–HOXA2 and HOXA1–HOXA5 heterodimerization appeared to be mainly nuclear (Figure 1F).

In summary, transcriptomic data indicated that, at a cellular level, *HOX* genes are largely co-expressed in the cells of the developing embryo. In many instances, HOX heterodimerization was reported in large-scale interactomic studies. Consistently, we validated the interaction between HOXA1, HOXA2, and HOXA5 proteins, showing their potential to form homo- and heterodimers that are mainly localized in the nucleus.

### 2.2. HOX Homeodomains Are Not Essential to Dimerization but Contribute to Nuclear Localization of the Interaction(s)

**Figure 2:**
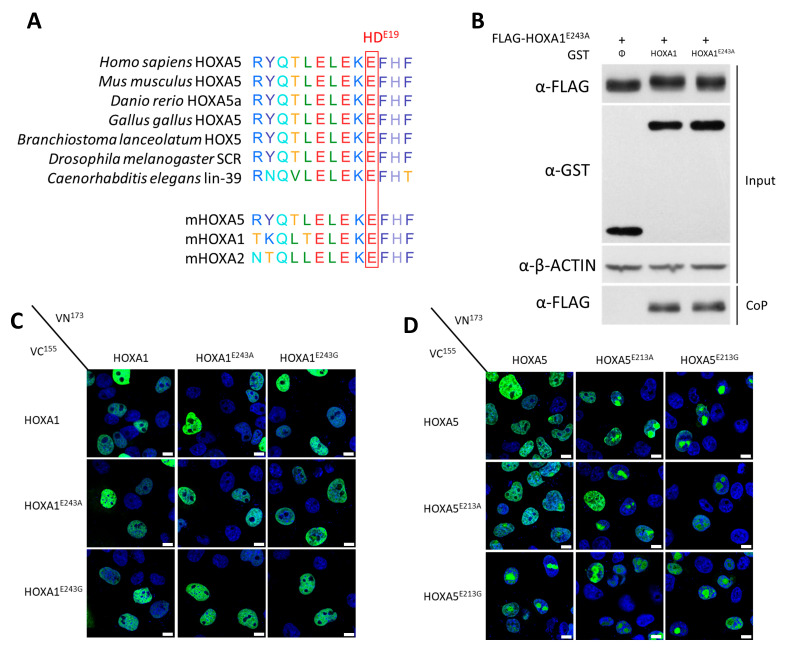
Impact of the E19A and E19G homeodomain mutations on HOXA1 and HOXA5 homodimerization. (**A**). **The E19 amino acid of the HD is conserved**. Amino acid alignment of the first α-helix of the homeodomain of drosophila SCR, SCR orthologs, murine HOXA5, murine HOXA1, and murine HOXA2. The conserved glutamate (E) residue at position 19 of the homeodomain is highlighted by a red rectangle. (**B**). **The E19 amino acid is not essential for HOXA1 dimerization**. Coprecipitation (CoP) assay. HEK293T cells were transfected with plasmids coding for FLAG-HOXA1^E243A^ in combination with GST, GST–HOXA1, or GST–HOXA1^E243A^. Proteins were analyzed by Western blotting before (Input) and after coprecipitation on glutathione beads (CoP). Antibodies were directed against GST or FLAG tags. β-ACTIN detection was used as the control. Presented blots are representative of at least three independent experiments (N ≥ 3). (**C**,**D**). The **E19 amino acid is not essential for HOXA1 and HOXA5 homodimerizations**. Bimolecular Fluorescence Complementation (BiFC). COS-7 cells were transfected with plasmids coding (**C**) for VC^155^HOXA1, VC^155^HOXA1^E243A^, or VC^155^HOXA1^E243G^ in combination with VN^173^HOXA1, VN^173^HOXA1^E243A^, or VN^173^HOXA1^E243G^, (**D**) for VC^155^HOXA5, VC^155^HOXA5^E213A^, or VC^155^HOXA5^E213G^ in combination with VN^173^HOXA5, VN^173^HOXA5^E213A^, or VN^173^HOXA5^E213G^. Upon interaction between the partner proteins, the VN^173^ and VC^155^ moieties of the Venus fluorescent protein are brought together and generate a green fluorescent signal. Nuclei were stained with DAPI (blue). Pictures were obtained using confocal microscopy. Scale bars = 10 μm. Presented pictures are representative of at least three independent experiments (N ≥ 3).

In the context of characterizing the homodimerization of the drosophila SCR protein, Papadopoulos et al. showed that the replacement of a glutamic acid (E) at position 19 of the HD by a glycine (E19G substitution) impaired the SCR–SCR interaction [16]. Since this residue is conserved within the HOX family among orthologs and paralogs (Figure 2A), we investigated if this amino acid could also be involved in murine HOXA5 (SCR homolog) and HOXA1 homodimerization. As glycine residues can impact the folding of α-helices [28], we also proceeded with a more neutral E-to-A substitution and compared E19G and E19A mutants with the wild-type proteins (WT).

The mutated HOX proteins were first tested for their transcriptional activity in a reporter assay with the *TSEII-luc* reporter in the presence of the TALE proteins PREP and PBX. *TSEII* is a HOX-responsive regulatory element derived from the somatostatin gene on which HOX–PBX dimers were shown to form [29]. All HOXA1 and HOXA5 mutant proteins failed to properly induce activation of the target reporter (Supplemental Figure S2A,B). Furthermore, loss of activity could not be attributed to the failure of nuclear import as mutant proteins were detected in the nucleus (Supplemental Figure S2C,D).

Thereafter, we assessed the homodimerization of the mutated versions of HOXA1 and HOXA5, fused either with FLAG and GST tags for coprecipitation or VN^173^ and VC^155^ for BiFC assays. Coprecipitation results supported that the HOXA1^E243A^ HD mutant did not lose its ability to form dimers with HOXA1^WT^ or HOXA1^E243A^ (Figure 2B). Definitive conclusions regarding the dimerization of HOXA1^E243G^, HOXA5^E213A^, and HOXA5^E213G^ could not be drawn as the abundance of these mutant versions was drastically lower than the WT, resulting in a coprecipitation output that was lower than the sensitivity threshold of the assay. Conversely, BiFC experiments yielded positive signals for all tested conditions (i.e., the test signal was at least three times stronger than the signal obtained in the control), indicating that none of these mutations led to a full disruption of the HOX–HOX complex (Figure 2C,D). Although the intensity of the BiFC signal varied among conditions, the quantitative impact of the mutations on dimerization ability could not be determined using this experimental procedure. This was primarily due to variations in mutant protein abundance compared to the WT, and the differing background signals present for the tested combinations.

**Figure 3:**
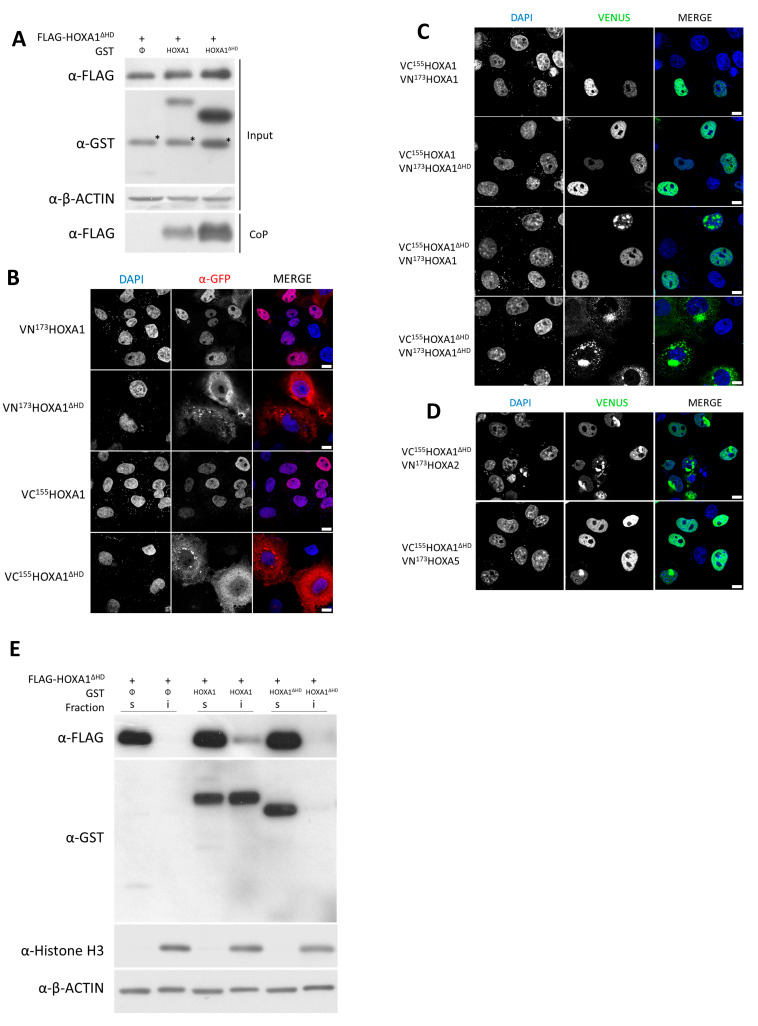
HOXA1 homeodomain is dispensable for dimerization but required for nuclear localization of the dimer. (**A**). **HOXA1 HD is not essential for dimerization**. Coprecipitation (CoP) assay. HEK293T cells were transfected with plasmids coding for FLAG–HOXA1^ΔHD^ in combination with GST, GST–HOXA1, or GST–HOXA1^ΔHD^. Proteins were analyzed by Western blotting before (Input) and after coprecipitation on glutathione beads (CoP). Antibodies were directed against GST or FLAG tags. β-ACTIN detection was used as the control. Provided blots are representative of at least three independent experiments (N ≥ 3). Since membranes with transferred proteins were repeatedly used for several protein detection assays, asterisks (*) indicate bands observed due to prior antibody incubations. (**B**). **HOXA1 HD is essential for HOXA1 localization**. Protein immunodetection. COS-7 cells were transfected with plasmids coding for VN^173^HOXA1, VC^155^HOXA1, VN^173^HOXA1^ΔHD^, or VC^155^HOXA1^ΔHD^ and subjected to immunofluorescence with anti-GFP antibodies (red). Nuclei were stained with DAPI (blue). Pictures were obtained using confocal microscopy. Scale bars = 10 μm. (**C**,**D**). **HOXA1 HD is essential for nuclear HOXA1 dimer localization**. Bimolecular Fluorescence Complementation (BiFC). COS-7 cells were transfected with plasmids coding (**C**) for VC^155^HOXA1 and VN^173^HOXA1^ΔHD^, VC^155^HOXA1 and VN^173^HOXA1 ^ΔHD^, VC^155^HOXA1^ΔHD^ and VN^173^HOXA1, or VC^155^HOXA1^ΔHD^ and VN^173^HOXA1^ΔHD^, (**D**) for VC^155^HOXA1^ΔHD^ and VN^173^HOXA2, or VC^155^HOXA1^ΔHD^ and VN^173^HOXA5. Upon interaction between the partner proteins, the VN^173^ and VC^155^ moieties of the Venus fluorescent protein are brought together and generate a green fluorescent signal. Nuclei were stained with DAPI (blue). Pictures were obtained using confocal microscopy. Scale bars = 10 μm. Presented pictures are representative of at least three independent experiments (N ≥ 3). (**E**). **HOXA1 HD is essential for HOXA1 chromatin localization**. Protein location in soluble and insoluble cell extracts. HEK293T cells were transfected with expression vectors coding for FLAG–HOXA1^ΔHD^ in combination with either GST, GST–HOXA1, or GST–HOXA1^ΔHD^. Proteins were extracted with IPLS buffer, and supernatants were recovered (s: soluble fraction). Pellets containing the insoluble proteins were then lysed with guanidium buffer (i: insoluble fraction). Western blotting was performed on both fractions with antibodies against FLAG, GST, Histone H3, and β-ACTIN.

Because a point mutation (i.e., E19) in the HD failed to disrupt the HOX–HOX interaction, we expanded the protein modification to the deletion of the entire HD (ΔHD). Both coprecipitation and BiFC showed a positive interaction between HOXA1–HOXA1^ΔHD^ and HOXA1^ΔHD^–HOXA1^ΔHD^, suggesting that the HD is not essential for HOXA1 homodimerization (Figure 3A,C). Similarly, BiFC experiments showed that the HOXA1 HD was not essential for heterodimerization with HOXA2 and HOXA5 (Figure 3D and Supplemental Figure S3A). Similar results were also obtained for the HOXA2^ΔHD^ variant (Supplemental Figure S3B).

While these results suggest that the HD is not critical to HOX dimerization, the impact of HD deletion on the distribution of the interaction has yet to be investigated. For the WT proteins, the two HOXA1 Venus fusions (VN^173^HOXA1 and VC^155^HOXA1) appeared to be mainly located in the nucleus, with minimal detection in the cytoplasm. Consistently, the localization of the homodimer was also observed mainly in the nucleus (Figure 3B,C). In contrast, for the HD-deprived proteins, VN^173^HOXA1^ΔHD^ and VC^155^HOXA1^ΔHD^, fusions displayed a mainly cytoplasmic localization and, consistently, an almost exclusive cytoplasmic localization of the homodimer signal (Figure 3B,C). Despite the observed cytoplasmic localization of VN^173^HOXA1^ΔHD^ and VC^155^HOXA1^ΔHD^ (Figure 3B), the BiFC signal associated with combinations of WT and ΔHD variants appeared to be primarily nuclear (VC^155^HOXA1-VN^173^HOXA1^ΔHD^ and VC^155^HOXA1^ΔHD^–VN^173^HOXA1, Figure 3C), suggesting that the presence of at least one functional HOXA1 HD is both necessary and sufficient for the dimer to occur in the nucleus. Similar results were obtained for heterodimer formation upon deletion of the HOXA2 HD, with the HOXA1–HOXA2^ΔHD^ heterodimer presenting a nuclear distribution and the HOXA1^ΔHD^–HOXA2^ΔHD^ heterodimer being detected in the cytoplasm (Supplemental Figure S3B).

Consistently with its function as a transcription factor, HOXA1 was detected in the nucleus (Figure 3B). Moreover, in cell fractionation assays, HOXA1 was detected both in the soluble protein fraction and in the insoluble protein fraction containing histones and DNA (i.e., the chromatin-associated protein fraction) upon cell lysis (Figure 3E, lane 3–4). As shown by immunofluorescence, deletion of the HD in HOXA1 led to cytoplasmic localization (Figure 3B) and detection of the deletant protein exclusively in the soluble fraction, suggesting that the HD was required for chromatin association of the protein (Figure 3E, lane 1–2). Interestingly, co-expression of HOXA1 together with HOXA1^ΔHD^ led to a relocation of HOXA1^ΔHD^ in the insoluble fraction, suggesting that the HOXA1–HOXA1^ΔHD^ complex localizes at the chromatin level (Figure 3E, lane 3–4).

Together, these results showed that the localization of HOX proteins both in the nucleus and at the chromatin level is HD-dependent. Moreover, our results support that the HD is not essential for dimerization, but at least one HD is required and sufficient for the homo- and heterodimers to localize in the nucleus. Finally, we showed that HOXA1^ΔHD^ localization at the chromatin level is increased in the presence of HOXA1^WT^, suggesting the formation of such complexes at the DNA level.

### 2.3. The Integrity of the Third Helix Is Crucial for the Nuclear Dimerization of HOXA1 Proteins

**Figure 4:**
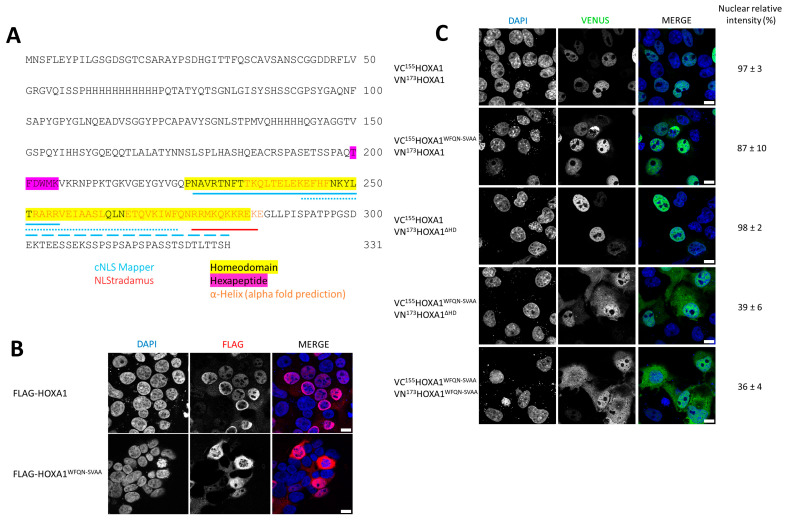
The integrity of the third helix of the homeodomain is important for the nuclear localization of HOX dimers. (**A**). **HOXA1 NLS predictions localize in the HD**. Protein sequence of HOXA1. Prediction of the nuclear localization signal (NLS) with two NLS prediction software, cNLS mapper (3 predicted NLSs: solid, dashed, and dotted blue lines) and NLStradamus (red). Alpha-fold prediction is from Uniprot (entry: P09022) (orange) (**B**). **The 3rd helix of HOXA1 is essential for HOXA1 nuclear localization**. Protein immunodetection. HEK293T cells were transfected with expression vectors for FLAG–HOXA1 or FLAG–HOXA1^WFQN-SVAA^ proteins and assayed by immunofluorescence with anti-FLAG (red). Nuclei were stained with DAPI (blue). Pictures were obtained using confocal microscopy. Scale bars = 10 μm. (**C**). **The 3rd helix of HOXA1 is essential for HOXA1 dimer nuclear localization**. Bimolecular Fluorescence Complementation (BiFC). COS-7 cells were transfected with plasmids coding for VC^155^HOXA1 and VN^173^HOXA1, VC^155^HOXA1^WFQN-SVAA^ and VN^173^HOXA1, VC^155^HOXA1 and VN^173^HOXA1^ΔHD^, VC^155^HOXA1^WFQN-SVAA^ and VN^173^HOXA1^ΔHD^, or VC^155^HOXA1^WFQN-SVAA^ and VN^173^HOXA1^WFQN-SVAA^. Upon interaction between the partner proteins, the VN^173^ and VC^155^ moieties of the Venus fluorescent protein are brought together and generate a green fluorescent signal. Nuclei were stained with DAPI (blue). Pictures were obtained using a confocal microscope. The proportion of the HOXA1-containing dimer signal that was nuclear vs. non-nuclear was calculated by comparing the signals in the green (Venus) and blue (DAPI) channels and expressed as the nuclear relative signal (%) ± relative error. Scale bars = 10 μm. Presented pictures are representative of at least three independent experiments (N ≥ 3).

The cytoplasmic localization observed for HOXA1^ΔHD^–HOXA1^ΔHD^ complexes (Figure 3B) suggests that the HD contains elements that are critical to nuclear dimerization of the protein. Nuclear localization signal (NLS) prediction software was therefore used to determine which amino acids might be involved in the process. These analyses highlighted potential NLS sequences overlapping with the HOXA1 HD, with cNLS mapper [30] flagging most of the HD as potentially important to nuclear import, and NLStradamus [31] predicting the involvement of the third helix of the HD, specifically (Figure 4A). Previous research reported that the HOXA1^WFQN-SVAA^ mutant, containing a four-amino acid mutation in the third helix of the HD, is predicted to be unable to bind DNA and is transcriptionally inactive [29]. In the present study, we showed that HOXA1^WFQN-SVAA^ mutant localization was also impaired, with a nuclear/cytoplasmic signal ratio that is lower than for HOXA1^WT^ (nuclear/total signal intensity: HOXA1^WT^ = 0.83, HOXA1^WFQN-SVAA^ = 0.61, Figure 4B). In addition, whereas the dimerization between VN^173^HOXA1 and VC^155^HOXA1^WFQN-SVAA^ was mainly detected in the nucleus, the dimerization of two mutant proteins, VN^173^HOXA1^WFQN-SVAA^ and VC^155^HOXA1^WFQN-SVAA^, was mainly observed in the cytoplasm (Figure 4C). Finally, HOXA1^WFQN-SVAA^ was still able to interact with HOXA1^ΔHD^ in the nucleus, but to a lower extent than with HOXA1^WT^ (relative nuclear signal intensity of 0.39 and 0.98, respectively; Figure 4C). These results support that the integrity of the third helix of the HOXA1 HD is crucial for the nuclear localization of the protein and the homodimer.

### 2.4. The HOXA2 Homeodomain Is Sufficient for Nuclear Homo- and Heterodimerization

**Figure 5:**
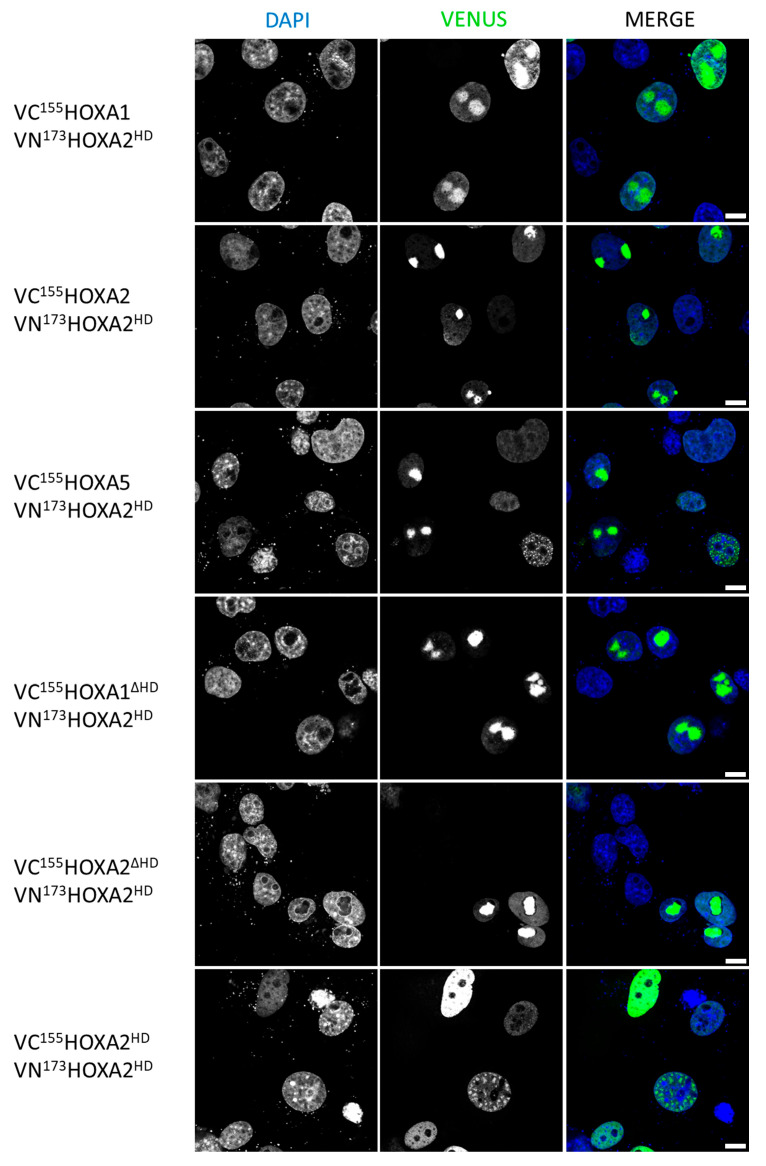
HOXA2 homeodomain is sufficient for dimer formation and nuclear localization of the dimer. Bimolecular Fluorescence Complementation (BiFC). COS-7 cells were transfected with plasmids coding for VN^173^HOXA2^HD^ and VC^155^HOXA1, VN^173^HOXA2^HD^ and VC^155^HOXA2, VN^173^HOXA2^HD^ and VC^155^HOXA5, VN^173^HOXA2^HD^ and VC^155^HOXA1^ΔHD^, VN^173^HOXA2^HD^ and VC^155^HOXA1^ΔHD^, VN^173^HOXA2^HD^ and VC^155^HOXA2^ΔHD^, or VN^173^HOXA2^HD^ and VN^173^HOXA2^HD^. Upon interaction between the partner proteins, the VN^173^ and VC^155^ moieties of the Venus fluorescent protein are brought together and generate a green fluorescent signal. Nuclei were stained with DAPI (blue). Pictures were obtained using confocal microscopy. Scale bars = 10 μm. Presented pictures are representative of at least three independent experiments (N ≥ 3).

Since the HD is conserved in HOX proteins and, based on the above results, appears to be involved in the nuclear localization of HOX homo- and heterodimers, the question arose whether the HD could be sufficient for nuclear dimerization. Because fusion peptides involving HOXA1^HD^ could not be detected upon cell transfection, we used HOXA2^HD^, which has high similarity (40 out of 60 amino acids are identical) and is more stable. The BiFC signal was observed in the nucleus when VN^173^HOXA2^HD^ was co-expressed with the full-length VC^155^HOXA1, VC^155^HOXA2, or VC^155^HOXA5 proteins, suggesting that the HD alone is sufficient to mediate dimeric interactions in the nucleus (Figure 5). VN^173^HOXA2^HD^ also presented a positive nuclear BiFC signal when co-expressed with VC^155^HOXA1^ΔHD^ or VC^155^HOXA2^ΔHD^ (Figure 5). Moreover, a positive BiFC signal was observed for the co-expression of VC^155^HOXA2^HD^ and VN^173^HOXA2^HD^ (Figure 5). Put together, these results suggest that at least two distinct HOX protein regions, the HD and a region outside of the HD, are sufficient to support dimerization, with neither region being strictly necessary to the process. Moreover, it appears that the presence of the HD is also sufficient for the nuclear localization of the dimer.

### 2.5. Non-HOX Homeodomain-Containing Proteins Are Not Sufficient for Nuclear Localization of HOX Heterodimers

HOX proteins are a subset of proteins belonging to a larger group of HD-containing proteins. Therefore, the non-HOX HD-containing proteins, PBX1A, MEIS1B, and DLX3, were tested for their ability to dimerize with full-length and HD-deleted versions of HOXA1 and HOXA2.

**Figure 6:**
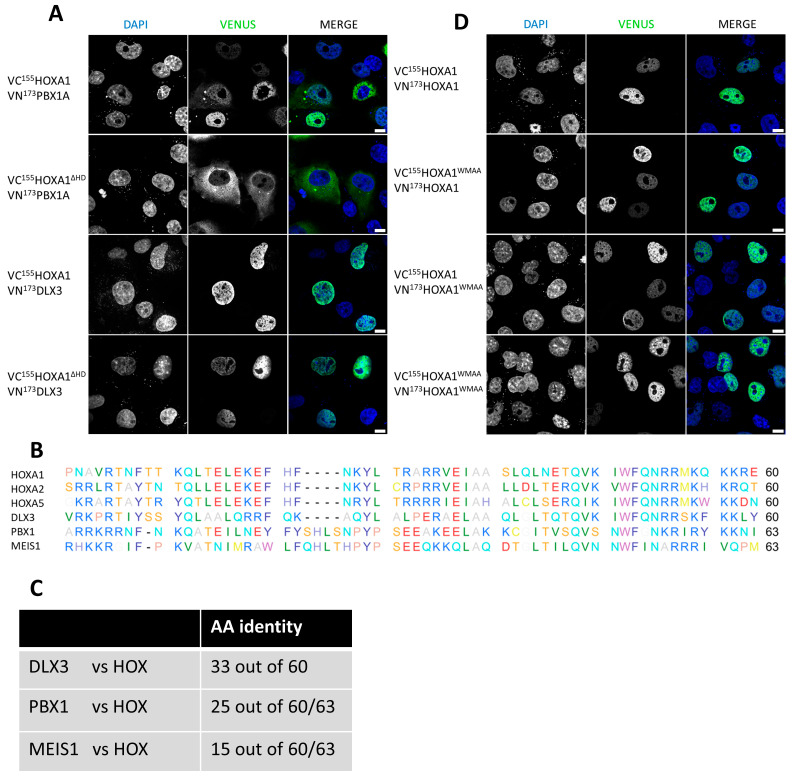
Non-HOX homeodomain proteins interact with HOX, but non-HOX homeodomains do not contribute to nuclear localization of the resulting HOX-containing dimers. HOXA1 HD is essential for PBX nuclear interaction. (**A**) Bimolecular Fluorescence Complementation (BiFC). COS-7 cells were transfected with plasmids coding for VN^173^PBX1A and either VC^155^HOXA1 or VC^155^HOXA1^ΔHD^, and VN^173^DLX3 and either VC^155^HOXA1 or VC^155^HOXA1^ΔHD^. Upon interaction between the partner proteins, the VN^173^ and VC^155^ moieties of the Venus fluorescent protein are brought together and generate a green fluorescent signal. Nuclei were stained with DAPI (blue). Pictures were obtained using confocal microscopy. Scale bars = 10 μm. Presented pictures are representative of at least three independent experiments (N ≥ 3). (**B**,**C**). Alignment of the amino acids of the homeodomain of HOXA1, HOXA2, HOXA5, HOXA9, DLX3, PBX1, and MEIS1 (**B**), and their identity (**C**). (**D**). **The hexapeptide is not essential for HOXA1 dimer nuclear localization**. Bimolecular Fluorescence Complementation (BiFC). COS-7 cells were transfected with plasmids coding for VN^173^HOXA1 and VC^155^HOXA1, VN^173^HOXA1 and VC^155^HOXA1^WMAA^, VN^173^HOXA1^WMAA^ and VC^155^HOXA1, or VN^173^HOXA1^WMAA^ and VC^155^HOXA1^WMAA^. Upon interaction between the partner proteins, the VN^173^ and VC^155^ moieties of the Venus fluorescent protein are brought together and generate a green fluorescent signal. Nuclei were stained with DAPI (blue). Pictures were obtained using confocal microscopy. Scale bars = 10 μm. Presented pictures are representative of at least three independent experiments (N ≥ 3).

In an effort to identify novel HOXA2 interactors, a yeast two-hybrid screen was previously conducted [32] and revealed DLX3 as a candidate HOXA2 interactor. DLX3 is potentially biologically relevant in light of the involvement of both HOXA2 and DLX3 in branchial arch patterning during embryogenesis [33,34]. Similarly to results obtained for HOX proteins, a BiFC signal was detected in the nucleus when VN^173^DLX3 was co-expressed with VC^155^HOXA1, VC^155^HOXA2, VC^155^HOXA1^ΔHD^, or VC^155^HOXA2^ΔHD^ (Figure 6A, Supplemental Figure S4A). In contrast, the well-known HOX interactor PBX1A presented a nuclear BiFC signal when fused to VN^173^ and co-expressed with VC^155^HOXA1, and a cytoplasmic signal in the presence of VC^155^HOXA1^ΔHD^. This indicates that despite containing an HD and being able to interact with HOX proteins, PBX1A was not capable of supporting an interaction with an HD-deficient HOX within the nucleus (Figure 6A). Concordantly, sequence alignment of HOX, DLX3, and PBX1 HDs revealed a higher degree of similarity between HOX and DLX3 than between HOX and PBX1 (Figure 6B,C).

Moreover, we investigated the impact of mutations in the HOXA1 hexapeptide, a motif critically involved in the interaction with PBX [35,36,37]. A nuclear signal was observed for co-expression of HOXA1^WMAA^ and either HOXA1^WT^ or HOXA1^WMAA^, suggesting that nuclear HOXA1 homodimerization is independent of the integrity of the hexapeptide and, therefore, the involvement of PBX (Figure 6D). We repeated the experiment and obtained similar results for HOXA2, suggesting that PBX1A and the integrity of the hexapeptide are non-essential for the nuclear dimerization of HOX (Supplemental Figure S4A,B). Interestingly, the fact that the PBX1A–HOX^ΔHD^ interaction was cytoplasmic (Figure 6A; Supplemental Figure S4A) while HOX–HOX^ΔHD^ dimerization was nuclear (Figure 3C) suggests that the HOX HD is required for its nuclear interaction with PBX1A.

Similar experiments carried out with MEIS1B also resulted in a nuclear BiFC signal associated with the VC^155^HOXA1–VN^173^MEIS1B interaction and a complete change to a cytoplasmic signal for the VC^155^HOXA1^ΔHD^–VN^173^MEIS1B interaction. While caution must be exerted with regards to these results due to the low reproducibility of the interaction between HOXA1 and MEIS1B (i.e., relatively small differences between the control and experimental conditions), these data support results obtained for PBX1, suggesting that the ability to interact with HOXA1^ΔHD^ in the nucleus is not a common feature of all HD-containing proteins (Supplemental Figure S4C).

## 3. Materials and Methods

### 3.1. Plasmid Constructs

Most reporter, expression, Gateway Entry (pEnt), Destination (pDest), or Expression (pExp) plasmids have been previously described (Supplemental Table S3). The entry vector for *DLX3* was obtained from hORFeome v3.1. The sequence coding for the wild-type *HOXA5* was PCR-amplified using primers reported in Supplemental Table S4 and inserted into a pDON223 vector using Gateway^®^ technology (Invitrogen, Waltham, MA, USA). The mutated versions of *HOXA1* and *HOXA5* were obtained by overlapping PCR using mutagenic primers listed in Supplemental Table S5. The resulting entry plasmids were confirmed by Sanger DNA sequencing. Expression plasmids were generated by LR reactions (Gateway^®^ technology, Invitrogen, Waltham, MA, USA) using various plasmid combinations listed in Supplemental Table S6.

### 3.2. Cell Culture and Transfection

HEK293T cells were cultured at 37 °C in a 5% CO_2_ atmosphere using DMEM medium (Thermo Fisher Scientific, MA, USA, #61965) supplemented with 10% fetal bovine serum (Thermo Fisher Scientific, Waltham, MA, USA, #10270-106), 100 U/mL of penicillin–streptomycin (Thermo Fisher Scientific, MA, USA, #15140122), and sodium pyruvate 1 mM (Thermo Fisher, Waltham, MA, USA, #11360-070). HEK293T cells were grown on polylysine-D-coated glass coverslips prior to the immunofluorescence assays. COS-7 cells were grown in DMEM medium (GIBCO, New York, NY, USA, #31885-023) supplemented with 10% fetal bovine serum and 100 U/mL of penicillin–streptomycin. Transfections of the indicated plasmid constructs were performed 24 h after plating using the JetPrime transfection reagent (Polyplus-transfection, Illkirch-Graffenstaden, France, #114-07) according to the manufacturer’s instructions.

### 3.3. Luciferase Assay

HEK293T cells were seeded on 24-well plates (175,000 cells per well) and transfected depending on the experimental condition, with 250 ng of *TSEII-luc*, 25 ng of the Renilla standard reporter plasmid, 50 ng of *PREP1*, 50 ng of *PBX1A*, and 100 ng of *HOX* expression vectors. The total amount of DNA was kept equal for all conditions by the addition of a carrier pCAT vector when required. After 48 h, the cells were harvested, and luciferase assays were performed following the manufacturer’s instructions (Dual-Glo^®^ Luciferase Assay System, Promega, Madison, WI, USA, #E2920).

### 3.4. Glutathione Coprecipitation

For this assay, 700,000 HEK293T cells were seeded per well of a 6-well plate and transfected with 1 μg of DNA (500 ng for the FLAG-tagged protein coding plasmid and 500 ng for the GST-tagged protein coding plasmid). The empty pDEST-GST vector was used as a negative control. Forty-eight hours after transfection, cells were rinsed with PBS and lysed in cold IPLS buffer (20 mM Tris-HCl pH 7.5, 120 mM NaCl, 0.5 mM EDTA, 0.5% NP40, and 10% glycerol) supplemented with 1× Complete™ protease inhibitor (Sigma, Burlington, VT, USA, #11873580001) for 20 min on ice with gentle agitation. Cell lysates were centrifuged for 5 min at 1000× *g* at 4 °C. Supernatants were collected and levels of FLAG- or GST-fused protein was analyzed by Western blotting using anti-FLAG (dilution: 1/5000, Sigma, Burlington, VT, USA, #F1804), anti-GST (dilution: 1/5000, Sigma, USA, #G1160), and anti-β-ACTIN (dilution: 1/20,000, Sigma, Burlington, VT, USA, #A3854) antibodies. Samples containing equal amounts of FLAG-tagged proteins were incubated with glutathione-sepharose beads (Sigma, Burlington, VT, USA, #GE17-0756-01) that were pre-washed with ice-cold IPLS lysis buffer. After overnight incubation on a rotating wheel at 4 °C, beads were washed three times with ice-cold IPLS then supplemented with Laemmli loading buffer for SDS-PAGE and boiled 5 min at 95 °C. Samples were centrifuged and analyzed by Western blotting (CoP samples). In parallel to protein coprecipitation, the abundance of fusion proteins engaged in the assay (Input samples) was analyzed by Western blotting as controls.

### 3.5. Cell Fractionation

HEK293T cells were transfected with 500 ng of indicated pExp plasmids encoding proteins fused with GST or FLAG. Forty-eight hours after transfection, proteins were extracted with IPLS buffer (20 mM Tris-HCl pH 7.5, 120 mM NaCl, 0.5 mM EDTA, 0.5% NP40, and 10% glycerol) supplemented with Complete™ protease inhibitor (Roche, Bâle, Switzerland, #11873580001) for 20 min on ice with gentle agitation. Cell lysates were collected and centrifuged for 5 min at 1000× *g* at 4 °C. The supernatant corresponding to the Western blots of soluble proteins was recovered. The pellets were washed once with IPLS and then lysed with Guanidium 6 M buffer (6 M Guanidium Hydrochloride, 500 mM NaCl, 20 mM Na_2_HPO^4^/NaH_2_PO^4^, pH 7.8). The protein level in both fractions was assessed by Western blotting using anti-FLAG (dilution: 1/5000, Sigma, Burlington, VT, USA, #F1804), anti-GST (dilution: 1/5000, Sigma, Burlington, VT, USA, #G1160), anti-HistoneH3 (dilution: 1/20,000, Sigma, Burlington, VT, USA, #07-690), and anti-β-ACTIN (dilution: 1/20,000, Sigma, Burlington, VT, USA, #A3854).

### 3.6. Bimolecular Fluorescence Complementation (BIFC)

For this assay, 50,000 COS-7 cells were cultured on glass coverslips and, 24 h after plating, were transfected with distinct combinations of pExpVN^173^ and pExpVC^155^ vectors for the fusion proteins and/or pDestVN^173^ and pDestVC^155^ empty controls, each at 500 ng. Twenty-four hours after transfection, cells were rinsed in PBS solution and fixed for 20 min with 4% paraformaldehyde (PFA) in PBS, then rinsed twice in TBS (50 mM Tris, 155 mM NaCl, pH 7.5) containing 0.1% Triton X-100. Coverslips were rinsed once in TB (50 mM Tris pH 7.5) containing 25 μg/mL of DAPI (4′-6-diamidino-2-phenylindole; Roche) prior to mounting in DAKO Fluorescent mounting medium (Agilent, SC, USA, S3023).

### 3.7. Immunofluorescence Assay

Transfected COS-7 or HEK293T cells cultured on glass coverslips were rinsed in PBS and fixed for 20 min with 4% PFA in PBS. Cells were further blocked with 10% low-fat milk in TBS-0.1% Triton X-100 solution for 30–45 min at RT, followed by overnight incubation in TBS-0.1% Triton X-100 1% low-fat milk solution at 4 °C, with mouse anti-FLAG (dilution: 1/150–1/200, Sigma, Burlington, VT, USA, #F1804,) or rabbit anti-GFP (dilution: 1/200, A11122, Invitrogen, Carlsbad, CA, USA) antibodies. Cells were rinsed three times for 10 min in TBS-0.1% Triton X-100 and incubated for 45 min at RT with Alexa Fluor^®^ 555 Anti-mouse IgG or Alexa Fluor^®^ 555 Anti-rabbit IgG (dilution: 1/500, Cell Signaling Technology, Danvers, MA, USA, #4409, #4413) in TBS-0.1% Triton X-100 solution. Cells were rinsed twice with TBS-0.1% Triton X-100 and once with TB (50 mM Tris pH 7.5) containing 25 μg/mL of DAPI (4′-6-diamidino-2-phenylindole, Roche, Bâle, Switzerland) prior to mounting in DAKO Fluorescent mounting medium (S3023, Agilent, Santa Clara, CA, USA).

### 3.8. Imaging

Glass coverslips were analyzed by epifluorescence (Axioskop 2, Zeiss, Oberkochen, Germany, magnification 10×) or confocal microscopy (Leica DMI8-Stellaris, White Laser-Diode 405, Leica Microsystems, Wetzlar, Germany, magnification 63×) using Las X software. Fluorescence signals were quantified using Fiji-ImageJ software (1.54f and v1.53k respectively). BiFC fluorescence from the test and the control conditions were quantified. The interaction was considered positive when the tested condition emitted at least 3 times more fluorescence than the 3 control conditions.

## 4. Discussion

HOX proteins exhibit a limited intrinsic DNA-binding specificity and it is thought that their activity is largely regulated by a panoply of context-specific interactions. Here, we showed that multiple HOX proteins have the ability to form homodimers or heterodimers with other HOX proteins, suggesting that these interactions may be a conserved feature common to mammalian HOX proteins. Based on the results presented above, we hypothesize that these dimerizations are a way to regulate subcellular protein localization, thereby affecting the ability of HOX protein to carry out transcriptional functions. These processes may therefore contribute to HOX specificity and could be a key element to solving the HOX paradox (i.e., HOX DNA binding specificity despite the high HD sequence similarity).

Consistently with their function as transcription factors, we showed that HOX proteins predominantly localize in the nucleus. Furthermore, the results presented here highlight that nuclear localization is dependent on the HD and its deletion leads to significantly altered protein distribution, namely more prevalent cytoplasmic localization. In particular, the third helix of the HD appears to be a crucial component of nuclear localization. However, results showing that HOXA1^WFQN-SVAA^ exhibits an intermediate cytoplasmic/nuclear localization pattern, with higher cytoplasmic distribution compared to HOXA1^WT^ but higher nuclear distribution than HOXA1^ΔHD^ (Figure 3B and Figure 4B), suggest that other determinants are also involved in HOXA1 nuclear localization. These observations underscore the critical role of the HD in nuclear localization, as reviewed earlier [38].

Our analysis of database records and results from our study suggest that HOX dimerization is a prevalent mode of interaction. Moreover, our BiFC analyses indicate that both HOX homo- and heterodimerization occur mainly within the nucleus, which confirms the results obtained in other studies, such as those reporting HOXA1–HOXA1, HOXA1–HOXD3, and SCR–SCR interactions [9,16,17].

The present study also evidenced that the HD is not essential for HOXA1 dimerization, with deletion of the HD in one or both interacting partners failing to disrupt complex formation. Conversely, the presence of at least one HD seems crucial for the nuclear localization of the dimer. Indeed, for HOXA1 proteins devoid of an HD, interactions predominantly occur in the cytoplasm (Figure 3C). However, while the HOXA1^ΔHD^–HOXA1^ΔHD^ dimer was cytoplasmic, the HOXA1^ΔHD^–HOXA1 dimer showed a strong nuclear signal (Figure 3C). Moreover, in light of the cytoplasmic distribution observed for HOXA1^ΔHD^ (Figure 3B), we can hypothesize that HOXA1 and possibly other HOX proteins are imported into the nucleus as dimers, with the presence of at least one HD being necessary for the nuclear transport of the complex; however, further investigations will have to be conducted to confirm this phenomenon.

Further insights regarding the role of the HD in nuclear localization can be drawn from results obtained for heterodimers between HOX and HD-containing TALE proteins. While HOXA1^ΔHD^ and PBX1A or MEIS1B heterodimers occurred mainly in the cytoplasm, both the HOXA1^ΔHD^–DLX3 and HOXA2^ΔHD^–DLX3 heterodimers were nuclear. When considering the higher sequence similarity between DLX3 and HOX HDs (ANTP class) compared to PBX1A and MEIS1B HDs (TALE class), these results support that the presence of at least one ANTP HD is required for the nuclear localization of HOX dimers.

While TALE proteins are important interactors of HOX proteins, their involvement in HOX dimerization has not been directly investigated here. However, we report that mutation of the hexapeptide motif, which is critical for the HOXA1–PBX interaction [35,36,37], does not impede HOX dimer formation in the nucleus, supporting that neither the hexapeptide nor PBX are essential for HOX dimer formation or for the nuclear localization of the dimers. Consistent with this observation, Phelan et al. demonstrated that two HOXA1 proteins are capable of binding to double-stranded DNA molecules in vitro, and that mutations in the hexapeptide did not disrupt the formation of these DNA-bound complexes [37]. Moreover, Fernandez et al. showed that the addition of PBX did not influence the interaction between HOXA1 and the truncated HOXA1(1-399). However, Mallen et al. reported that the addition of PBX and MEIS can enhance the interaction between HOXA2 and HOXA3 [15]. On the other hand, it is conceivable that HOX dimerization could compete with HOX–TALE interactions. In this context, it has been demonstrated that HOXA1(1-399) decreases HOXA1–PBX binding at the DNA level, as well as their transcriptional activity in a luciferase assay [26]. The potential interplay between HOX–HOX and HOX–TALE dimers at enhancers, whether synergistic or antagonistic, therefore warrants further investigation.

Consistent with the possible involvement of the HD in the trafficking of HOX dimers (e.g., a nuclear signal for HOXA1–HOXA1^ΔHD^ versus a cytoplasmic signal for HOXA1^ΔHD^–HOXA1^ΔHD^), we provided evidence that HOXA1^ΔHD^ is relocated to the chromatin-containing (insoluble) fraction in the presence of HOXA1^WT^. These data suggest that a wild-type HOXA1 protein with an intact HD can influence the localization of a HOXA1 derivative lacking the HD, which, by itself, is undetected in the chromatin fraction. This also supports a potential direct association between HOXA1 dimers and DNA; however, further investigation will be required to determine whether the HOX transcription factors interact as homo- or heterodimers in solution or are bound to DNA. With regards to the identification of protein–protein interactions at the chromatin in cellulo, a significant limitation of the coprecipitation experiments used in this study is that the pull-down is conducted on soluble proteins. Indeed, the non-denaturing conditions required to preserve protein–protein interactions during cell lysis and protein extraction maintain DNA and strongly DNA-bound proteins (e.g., histones) in the insoluble fraction [39]. This limits the scope of such interactomic experiments to protein interactions which are not associated with DNA, or only weakly bound to DNA. As shown here, a non-negligible proportion of HOX proteins remains in the insoluble fraction (Figure 3E and Supplemental Figure S5) and are de facto excluded from coprecipitation assays. Although BiFC analyses can provide some insights regarding interactions occurring in the nucleus, the resolution is insufficient to establish the association of protein complexes with DNA.

In this regard, the ability of HOX complexes to cooperatively bind DNA has been previously reported for UBX and SCR homodimerizations [16,17,22], as well as for HOXB2–HOXB13 and HOXD12–HOXA3 heterodimerizations [19]. It has also been demonstrated that HOXA2 and HOXA3 bind common genomic regions in the developing mouse embryo, but it is not yet known whether they bind these sites together or if the binding is mutually exclusive [24]. In addition, although HOXA2 and HOXA3 were shown to form complexes in HEK293T cells, no synergic activity was observed when combining HOXA2 and HOXA3 and no DNA binding of the heterodimer was reported [15,24]. As suggested by Mallen et al. for the HOXA2–HOXA3 complex and potentially applicable to other HOX proteins due to the widespread nature of the dimerization processes within the protein family, the formation of HOX heterodimers could provide an explanation for the “posterior prevalence” phenomenon. Posterior prevalence is a property of HOX TFs characterized by the dominance of the function of proteins expressed at later embryonic stages, which are absent in anterior territories, over those expressed at earlier stages. Indeed, *HOX* gene knock-outs generally exhibit defects in their most anterior domains of expression (where more posterior genes are not expressed), leaving the identity of more posterior territories (where more posterior genes become active) unchanged (reviewed by [40]). According to this hypothesis, anterior HOX would be trapped by more posterior HOX proteins, thereby inhibiting their association with DNA and limiting their transcriptional activity. Posterior HOX proteins would therefore act as dominant negatives, capable of initiating their differentiation program in their most anterior domain of expression.

In contrast with what has been reported for SCR, our data indicate that mutation of the 19th residue in the HD does not abolish homodimerization. In fact, data obtained with the HD-deleted HOX variant and the HOX HD alone indicated that while the HD is sufficient for HOX–HOX interactions to occur, it is not strictly necessary. In turn, this supports the involvement of at least two HOX domains, the HD among them, in the interaction. This conclusion is supported by other studies, such as those showing that HOXA1 HD is not essential for dimerization [26] and that the HD of the drosophila HOX homolog UBX is insufficient for the cooperative binding of UBX to DNA [21]. Furthermore, another study reporting an interaction between two HOX proteins from the nematode *Caenorhabditis elegans*, LIN-39 and MAB-5, showed that a mutation in the C-terminal part of LIN-39 (while not affecting the HD) led to a disruption of its interaction with MAB-5, indicating that regions outside of the HD might also be involved in the interaction.

Finally, the results presented here underscore the complex and nuanced dynamics of HOX dimerization processes. Previous studies investigating combinatorial *HOX* mutations revealed, in many cases, phenotypes that were unexpected on the basis of the observed effects of single-gene mutations (reviewed by [41]). Genetic interactions other than the redundancy between paralogs have also been highlighted between *HOX* genes supporting possible complex interplays between these genes [42]. The molecular mechanisms involved in such interplays have barely been explored, and the hypothesis that HOX protein homo- and heterodimerization could be key to understanding these genetic networks warrants further investigation.

In conclusion, the present findings support that the homo- and heterodimerization of HOX proteins are a shared and common feature within the family. Moreover, in light of the impact of these processes on the subcellular distribution of HOX proteins, it appears that dimerization might serve as a regulatory mechanism to control HOX-dependent transcriptional activity, adding a new layer to the already complex cross-regulatory network of *HOX* genes. HOX dimerization could be key to better understanding (i) the HOX protein paradox phenomenon by partially explaining the protein DNA-binding specificity in the face of high HD sequence similarity, (ii) the complex genetic interaction observed in vivo, or (iii) the posterior prevalence paradigm. Given the extensive co-expression of *HOX* genes in embryonic territories and at the cellular level and the increasing evidence of protein interactions, further investigation—notably regarding the mode of action and functional consequences of these interactions—could provide key insights into mechanisms crucial to shaping animal embryos while potentially shedding light on HOX involvement in pathologies such as cancer.

## Data Availability

Data is contained within the article or Supplementary Material.

## Supplementary Materials

The following supporting information can be downloaded at: https://www.mdpi.com/article/10.3390/ijms26010423/s1.
