# Supplementary material for: Homeodomain Involvement in Nuclear HOX Protein Homo- and Heterodimerization
Source: Int J Mol Sci. 2025 Jan 6;26(1):423. doi: 10.3390/ijms26010423 (PMC11721573; doi:10.3390/ijms26010423)
Supplement: Supplementary file 1 [file ijms-26-00423-s001.zip › ijms-3396469-Supplemental Figures.pdf]

Supplemental Materials—Figures

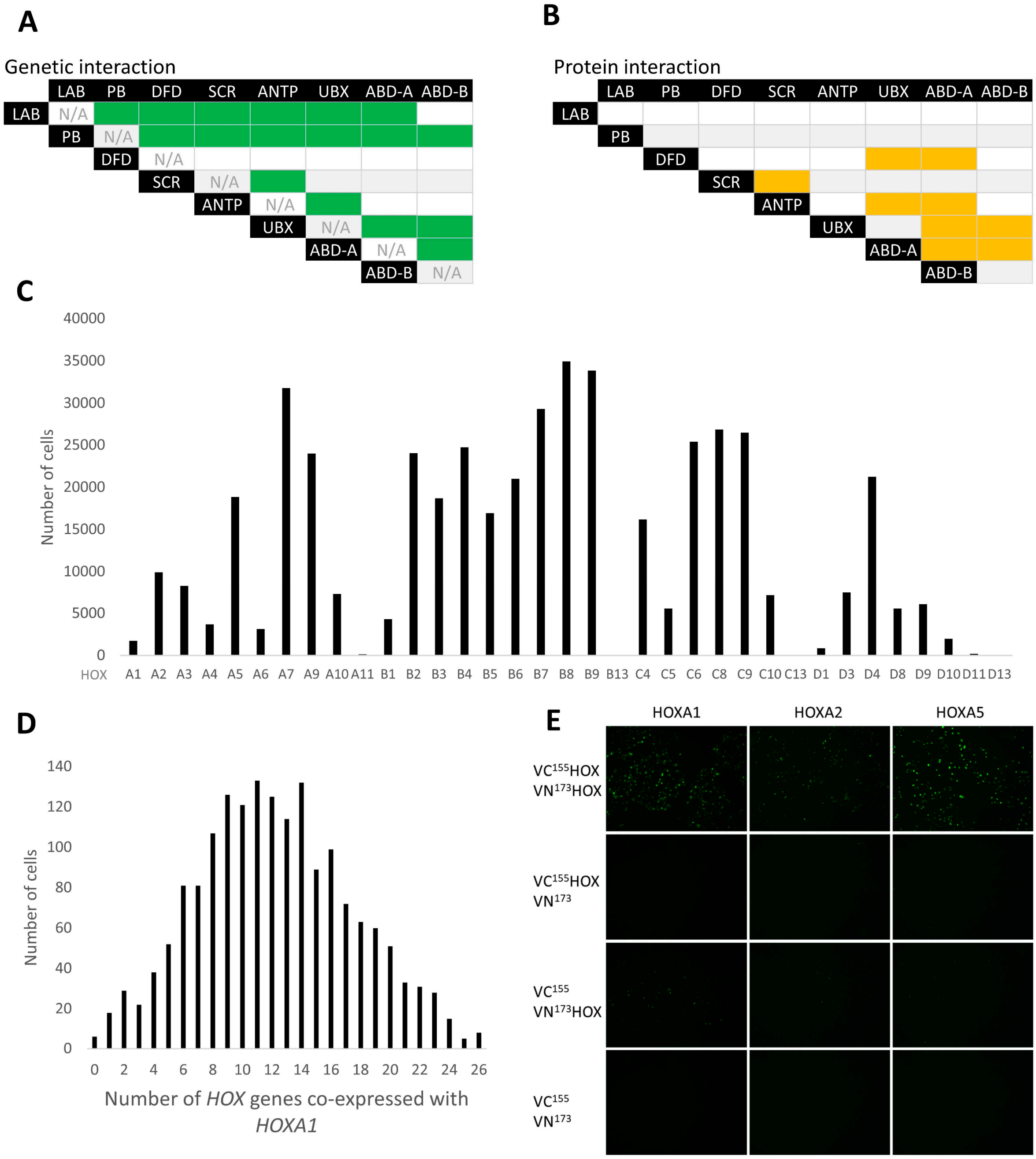

Figure S1. Interaction between HOX proteins is widely shared. **A–B**: Reported drosophila HOX protein and genetic interactions. Compilation of HOX-HOX genetic and protein interactions in *D. melanogaster* reported in the BIOGRID database. Green and yellow boxes in the double-entry table respectively indicate genetic (**A**) and protein (**B**) interactions. N/A = Not Applicable. **C–D**: *HOX* are co-expressed. Single-cell RNA sequencing data were extracted from [1], using normalized counts. During the development of the spinal cord, *HOX* genes are largely co-expressed. Graphical representation of the number of cells expressing each *HOX* gene (**C**). Visualization of *HOX* gene co-expression in *HOXA1* expressing cells (**D**). **E**: HOX homodimerize. Bimolecular Fluorescence Complementation (BiFC). COS-7 cells were transfected with plasmids coding for VC^{155}HOXA1 and VN^{173}HOXA1, VC^{155}HOXA2 and VN^{173}HOXA2, or VC^{155}HOXA5 and VN^{173}HOXA5. Upon interaction between the partner proteins, the VN^{173} and VC^{155} moieties of the Venus fluorescent protein are brought together and generate a green, fluorescent signal. Controls consisted in co-expression of each HOX-fusion protein with its complementary unfused Venus fragment (VC^{155}HOX with VN^{173}, VC^{155} with VN^{173}HOX) or combination of both unfused VN^{173} and VC^{155} Venus polypeptides. Interactions were considered validated when the signal was three times superior to each VN^{173} and VC^{155} controls. For the subsequent BiFC experiments, control conditions are not shown but were similar to those shown here, interactions were considered validated using the same criteria.

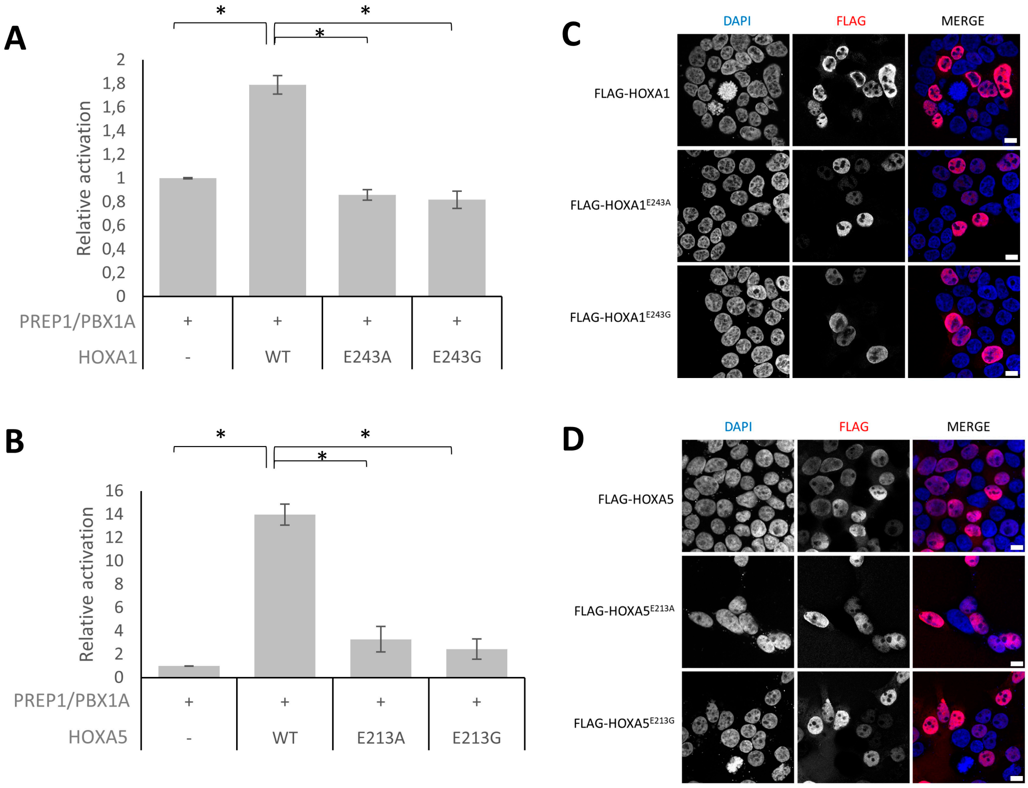

Figure S2. Impact of the E19A and E19G homeodomain mutations in HOXA1 (**A**,**C**) and HOXA5 (**B**,**D**). **A**–**B**: The E19 amino acid of the HD is essential for HOX transcriptional activity. Activity assays.: HEK293T cells were transfected with a *TSEII-luc* reporter and expression vectors for the indicated FLAG-HOX proteins. The interactors PREP and PBX were also transfected in all conditions. A constitutively active luciferase expression vector (pGL4.74 coding for *hRluc*) was included in each transfection for assay normalisation. The data are presented as *TSEII-luc*/*hRluc* activity ratios and are representative of three biological replicates. Pairwise comparisons using t-tests were performed (* = p-value < 0.05, adjusted p-value = Holm-Bonferroni). **C**–**D**: The E19 amino acid of the HD is not essential for nuclear HOX localization. HEK293T cells were transfected with expression vectors for the indicated HOX proteins and subjected to immunofluorescence with anti-FLAG antibodies (red). Nuclei were stained with DAPI (blue). Pictures were obtained using confocal microscopy. Scale bars = 10 µm. Presented pictures are representative of at least three independent experiments ($N \geq 3$).

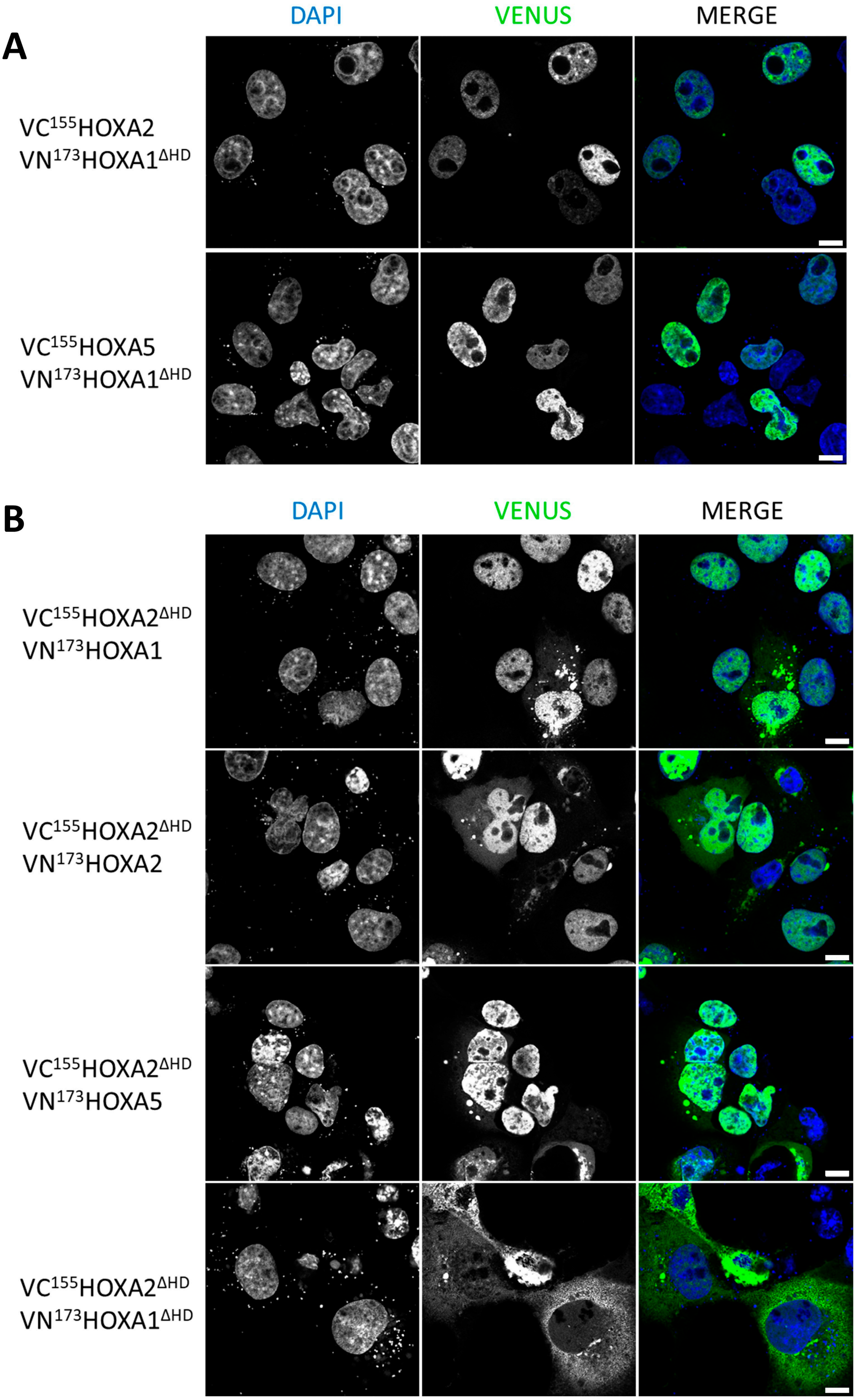

Figure S3. The homeodomain is not essential for HOX dimerization, but necessary for nuclear localisation of the dimer. **A**–**B**: Bimolecular Fluorescence Complementation (BiFC). COS-7 cells were transfected with plasmids coding (**A**) for VC^{155}HOXA2, or VC^{155}HOXA5 and VN^{173}HOXA1$^{\Delta HD}$, (**B**) for VC^{155}HOXA2$^{\Delta HD}$ and VN^{173}HOXA1, VN^{173}HOXA2, VN^{173}HOXA5, or VN^{173}HOXA1$^{\Delta HD}$. Upon interaction between the partner proteins, the VN^{173} and VC^{155} moieties of the Venus fluorescent protein are brought together and generate a green, fluorescent signal. Nuclei were stained with DAPI (blue). Pictures were obtained using confocal microscopy. Scale bars = 10 μm. Presented pictures are representative of at least three independent experiments ($N \geq 3$).

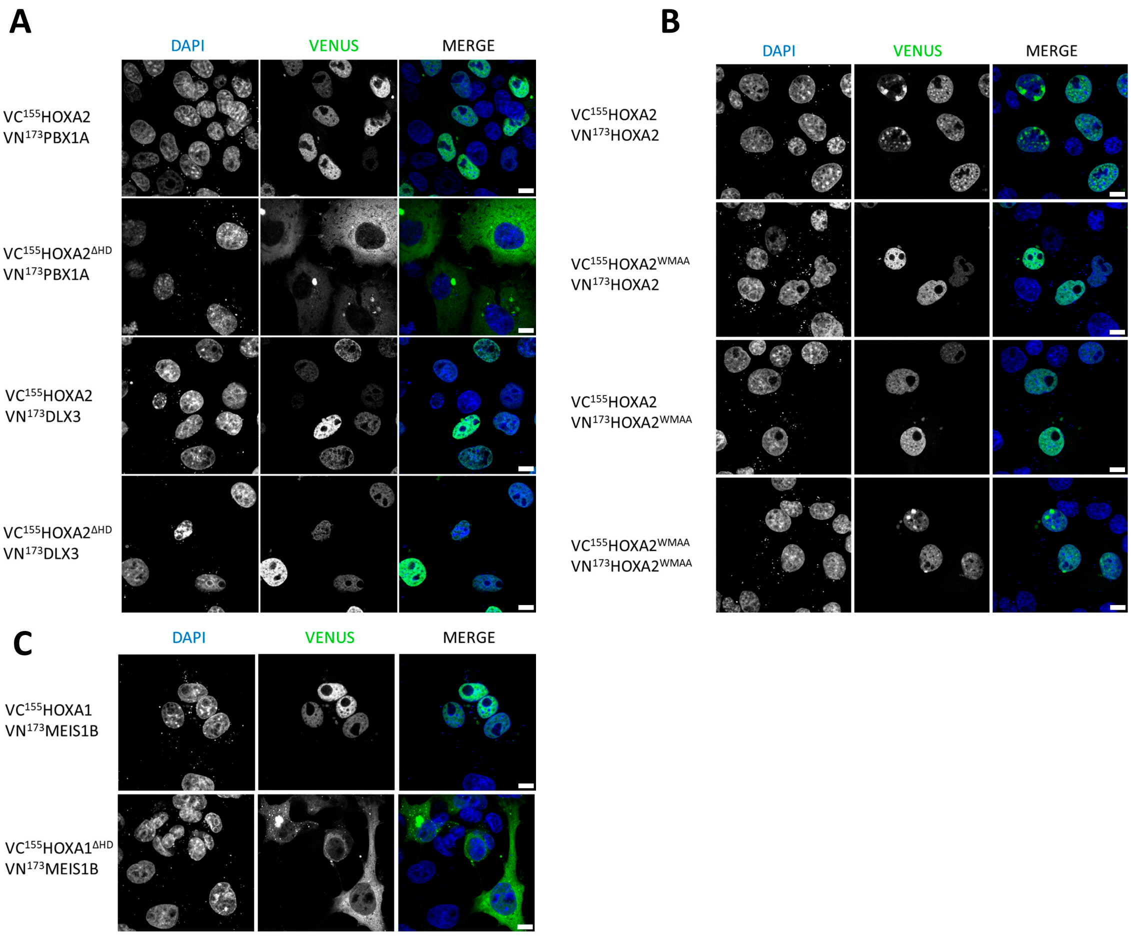

Figure S4. Non-HOX homeodomain proteins interact with HOX, but non-HOX homeodomains do not contribute to nuclear localisation of the resulting HOX-containing dimers. **A**–**B**: HOXA2 HD is essential for PBX nuclear interaction, but the hexapeptide is not essential for HOXA2 dimer nuclear localization. Bimolecular Fluorescence Complementation (BiFC). COS-7 cells were transfected with plasmids coding (**A**) for VN^{173}PBX1A and either VC^{155}HOXA2 or VC^{155}HOXA2$^{\Delta HD}$, VN^{173}DLX3 and either VC^{155}HOXA2 or VC^{155}HOXA2$^{\Delta HD}$, (**B**) for VN^{173}HOXA2 and VC^{155}HOXA2, VN^{173}HOXA2 and VC^{155}HOXA2^{WMAA}, VN^{173}HOXA2^{WMAA} and VC^{155}HOXA2 or VN^{173}HOXA2^{WMAA} and VC^{155}HOXA2^{WMAA}, (**C**) for VN^{173}MEIS1B and either VC^{155}HOXA1 or VC^{155}HOXA1$^{\Delta HD}$. Upon interaction between the partner proteins, the VN^{173} and VC^{155} moieties of the Venus fluorescent protein are brought together and generate a green, fluorescent signal. Nuclei were stained with DAPI (blue). Pictures were obtained using confocal microscopy. Scale bars = 10 µm. Presented pictures are representative of at least three independent experiments ($N \geq 3$).

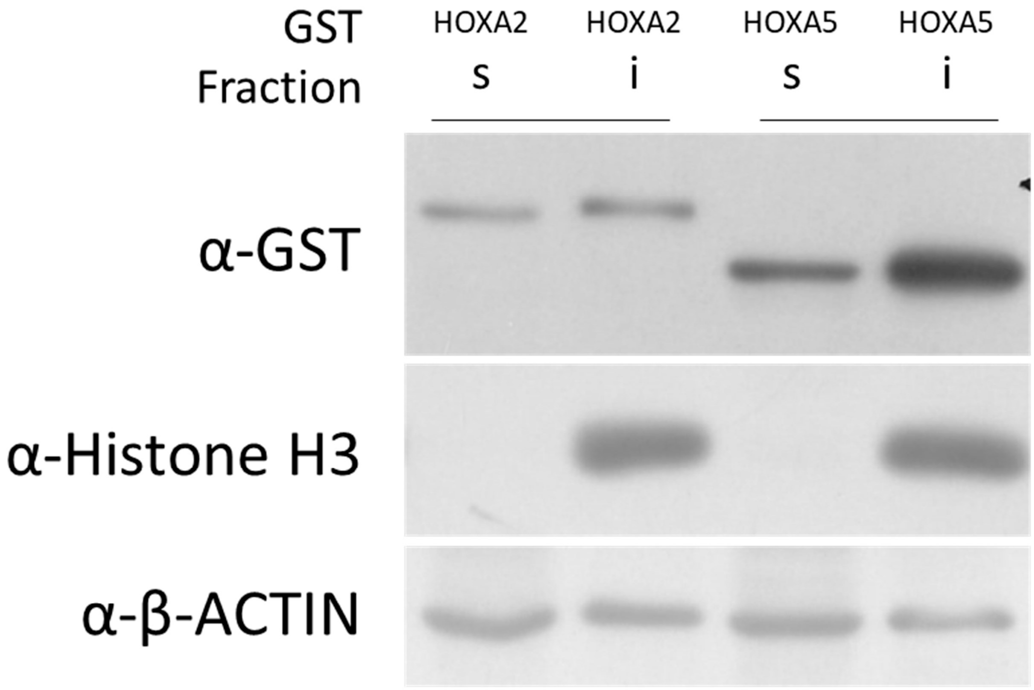

Figure S5. HOX proteins are detected in both the soluble and insoluble fractions of HEK293T cell extract. HOXA2 and HOXA5 detection in soluble and insoluble cell extracts. HEK293T cells were transfected with expression vectors coding for GST-HOXA2 and GST-HOXA5. Proteins were extracted with IPLS buffer and supernatants were recovered (s: soluble fraction). Pellets containing in-soluble proteins were then lysed with guanidium buffer (i: insoluble fraction). Western blottings were performed on both fractions with antibodies against GST, Histone H3 and β-ACTIN.

References

1. Delile, J.; Rayon, T.; Melchionda, M.; Edwards, A.; Briscoe, J.; Sagner, A. Single cell transcriptomics reveals spatial and temporal dynamics of gene expression in the developing mouse spinal cord. *Development* **2019**, *146*, dev173807. https://doi.org/10.1242/dev.173807.
