# Supplementary material for: Homeodomain Involvement in Nuclear HOX Protein Homo- and Heterodimerization
Source: Int J Mol Sci. 2025 Jan 6;26(1):423. doi: 10.3390/ijms26010423 (PMC11721573; doi:10.3390/ijms26010423)
Supplement: Supplementary file 1 [file ijms-26-00423-s001.zip › ijms-3396469-Supplemental Tables.pdf]

Supplemental Table S1. mouse and human HOX-HOX interactions reported in the BioGRID database.

Interactor A	Interactor A Species	Interactor B	Interactor B Species	Experimental Evidence Code	Role	Dataset	Throughput	Curated By
HOXA1	*H. sapiens*	HOXA1	*H. sapiens*	PCA	HIT	Taminiau A (2016)	Low	BioGRID
HOXA1	*H. sapiens*	HOXA1	*H. sapiens*	Two-hybrid	HIT	Luck K (2020)	High	BioGRID
HOXA1	*H. sapiens*	HOXA1	*H. sapiens*	Two-hybrid	HIT	Rolland T (2014)	High	BioGRID
HOXA1	*H. sapiens*	HOXA1	*H. sapiens*	Two-hybrid	HIT	Yu H (2011)	High	BioGRID
HOXA1	*H. sapiens*	HOXA1	*M. musculus*	PCA	BAIT	Lambert B (2012)	High	BioGRID
HOXA1	*H. sapiens*	HOXA1	*M. musculus*	Two-hybrid	BAIT	Lambert B (2012)	Low/High	BioGRID
HOXA1	*H. sapiens*	HOXB1	*H. sapiens*	Affinity Capture-MS	HIT	Huttlin EL (2021)	High	BioGRID
HOXA1	*H. sapiens*	HOXB9	*H. sapiens*	Two-hybrid	HIT	Luck K (2020)	High	BioGRID
HOXA1	*M. musculus*	HOXD3	*H. sapiens*	Affinity Capture-Western	BAIT	Lambert B (2012)	High	BioGRID
HOXA1	*M. musculus*	HOXD3	*H. sapiens*	PCA	HIT	Lambert B (2012)	High	BioGRID
HOXA1	*M. musculus*	HOXD3	*H. sapiens*	Two-hybrid	HIT	Lambert B (2012)	Low/High	BioGRID
HOXA2	*M. musculus*	HOXA10	*M. musculus*	Co-fractionation	HIT	Pourhaghighi R (2020)	High	BioGRID
HOXA2	*M. musculus*	HOXA4	*M. musculus*	Co-fractionation	BAIT	Pourhaghighi R (2020)	High	BioGRID
HOXA2	*M. musculus*	HOXA5	*M. musculus*	Co-fractionation	BAIT	Pourhaghighi R (2020)	High	BioGRID
HOXA2	*M. musculus*	HOXB4	*M. musculus*	Co-fractionation	BAIT	Pourhaghighi R (2020)	High	BioGRID
HOXA2	*M. musculus*	HOXD10	*M. musculus*	Co-fractionation	BAIT	Pourhaghighi R (2020)	High	BioGRID
HOXA4	*M. musculus*	HOXA5	*M. musculus*	Co-fractionation	HIT	Pourhaghighi R (2020)	High	BioGRID
HOXA4	*M. musculus*	HOXB4	*M. musculus*	Co-fractionation	HIT	Pourhaghighi R (2020)	High	BioGRID
HOXA5	*H. sapiens*	HOXB7	*H. sapiens*	Affinity Capture-MS	HIT	Huttlin EL (2021)	High	BioGRID
HOXA5	*H. sapiens*	HOXD4	*H. sapiens*	Affinity Capture-MS	HIT	Huttlin EL (2021)	High	BioGRID
HOXA5	*M. musculus*	HOXA10	*M. musculus*	Co-fractionation	HIT	Pourhaghighi R (2020)	High	BioGRID
HOXA5	*M. musculus*	HOXB4	*M. musculus*	Co-fractionation	HIT	Pourhaghighi R (2020)	High	BioGRID
HOXA7	*H. sapiens*	HOXB7	*H. sapiens*	Affinity Capture-MS	HIT	Huttlin EL (2021)	High	BioGRID
HOXA10	*M. musculus*	HOXD10	*M. musculus*	Co-fractionation	BAIT	Pourhaghighi R (2020)	High	BioGRID
HOXB1	*H. sapiens*	HOXD1	*H. sapiens*	Affinity Capture-MS	BAIT	Huttlin EL (2021)	High	BioGRID

HOXB2	*H. sapiens*	HOXB2	*H. sapiens*	Two-hybrid	HIT	Wang XW (2023)	High	BioGRID
HOXB4	*H. sapiens*	HOXD10	*M. musculus*	Co-fractionation	BAIT	Pourhaghighi R (2020)	High	BioGRID
HOXB5	*H. sapiens*	HOXC4	*H. sapiens*	Affinity Capture-MS	HIT	Huttlin EL (2015)	High	BioGRID
HOXB5	*H. sapiens*	HOXC4	*H. sapiens*	Affinity Capture-MS	HIT	Huttlin EL (2017)	High	BioGRID
HOXB5	*H. sapiens*	HOXC4	*H. sapiens*	Affinity Capture-MS	HIT	Huttlin EL (2021)	High	BioGRID
HOXB13	*H. sapiens*	HOXD4	*H. sapiens*	Two-hybrid	BAIT	Ravasi T (2010)	High	BioGRID
HOXC8	*H. sapiens*	HOXD3	*H. sapiens*	Two-hybrid	HIT	Luck K (2020)	High	BioGRID
HOXC9	*H. sapiens*	HOXC13	*H. sapiens*	Two-hybrid	HIT	Ravasi T (2010)	High	BioGRID
HOXC9	*H. sapiens*	HOXD1	*H. sapiens*	Two-hybrid	HIT	Ravasi T (2010)	High	BioGRID
HOXC9	*H. sapiens*	HOXD9	*H. sapiens*	Two-hybrid	HIT	Ravasi T (2010)	High	BioGRID

[12, 28, 50-56]

Supplemental Table S2. drosophila HOX-HOX interactions reported in the BioGRID database.

Interactor A	Interactor A Species	Interactor B	Interactor B Species	Experimental Evidence	Role	Dataset	Throughput	Curated By
LAB	D. melanogaster	ABD-A	D. melanogaster	Phenotypic Suppression	HIT	Hirth F (2001)	Low	FlyBase
LAB	D. melanogaster	ANTP	D. melanogaster	Phenotypic Suppression	HIT	Hirth F (2001)	Low	FlyBase
LAB	D. melanogaster	DFD	D. melanogaster	Phenotypic Suppression	HIT	Hirth F (2001)	Low	FlyBase
LAB	D. melanogaster	SCR	D. melanogaster	Phenotypic Suppression	HIT	Hirth F (2001)	Low	FlyBase
LAB	D. melanogaster	UBX	D. melanogaster	Phenotypic Suppression	HIT	Hirth F (2001)	Low	FlyBase
PB	D. melanogaster	SCR	D. melanogaster	Phenotypic Suppression	HIT	Boube M (2000)	Low	FlyBase
PB	D. melanogaster	SCR	D. melanogaster	Phenotypic Suppression	BAIT	Percival-Smith A (1999)	Low	FlyBase
PB	D. melanogaster	SCR	D. melanogaster	Phenotypic Suppression	BAIT	Percival-Smith A (2005)	Low	FlyBase
PB	D. melanogaster	ABD-A	D. melanogaster	Phenotypic Suppression	BAIT	Percival-Smith A (2005)	Low	FlyBase
PB	D. melanogaster	ABD-B	D. melanogaster	Phenotypic Suppression	BAIT	Percival-Smith A (2005)	Low	FlyBase
PB	D. melanogaster	ANTP	D. melanogaster	Phenotypic Suppression	BAIT	Percival-Smith A (2005)	Low	FlyBase
PB	D. melanogaster	DFD	D. melanogaster	Phenotypic Suppression	BAIT	Percival-Smith A (2005)	Low	FlyBase
PB	D. melanogaster	LAB	D. melanogaster	Phenotypic Suppression	BAIT	Hirth F (2001)	Low	FlyBase
PB	D. melanogaster	UBX	D. melanogaster	Phenotypic Suppression	BAIT	Percival-Smith A (2005)	Low	FlyBase
DFD	D. melanogaster	ABD-A	D. melanogaster	PCA	BAIT	Bischof J (2018)	Low	FlyBase

DFD	D. melanogaster	UBX	D. melanogaster	PCA	BAIT	Bischof J (2018)	Low	FlyBase
SCR	D. melanogaster	SCR	D. melanogaster	Affinity Capture-Western	HIT	Papadopoulos DK (2012)	Low	FlyBase
SCR	D. melanogaster	SCR	D. melanogaster	PCA	HIT	Papadopoulos DK (2012)	Low	FlyBase
SCR	D. melanogaster	SCR	D. melanogaster	PCA	HIT	Papadopoulos DK (2015)	Low	FlyBase
SCR	D. melanogaster	ANTP	D. melanogaster	Phenotypic Suppression	BAIT	Kennison JA (1988)	Low	FlyBase
ANTP	D. melanogaster	UBX	D. melanogaster	PCA	BAIT	Bischof J (2018)	Low	FlyBase
ANTP	D. melanogaster	UBX	D. melanogaster	Phenotypic Enhancement	HIT	HANNAH-ALAVA A (1964)	Low	FlyBase
ANTP	D. melanogaster	UBX	D. melanogaster	Phenotypic Suppression	HIT	Li X (1999)	Low	FlyBase
ANTP	D. melanogaster	ABD-A	D. melanogaster	PCA	BAIT	Bischof J (2018)	Low	FlyBase
ANTP	D. melanogaster	UBX	D. melanogaster	PCA	BAIT	Bischof J (2018)	Low	FlyBase
ANTP	D. melanogaster	UBX	D. melanogaster	Phenotypic Enhancement	HIT	HANNAH-ALAVA A (1964)	Low	FlyBase
ANTP	D. melanogaster	UBX	D. melanogaster	Phenotypic Suppression	HIT	Li X (1999)	Low	FlyBase
UBX	D. melanogaster	ABD-A	D. melanogaster	PCA	BAIT	Hudry B (2011)	Low	FlyBase
UBX	D. melanogaster	ABD-A	D. melanogaster	Phenotypic Suppression	HIT	Casares F (1996)	Low	FlyBase
UBX	D. melanogaster	ABD-A	D. melanogaster	Phenotypic Suppression	HIT	de Navas L (2006)	Low	FlyBase
UBX	D. melanogaster	ABD-B	D. melanogaster	PCA	HIT	Bischof J (2018)	Low	FlyBase
UBX	D. melanogaster	ABD-B	D. melanogaster	Phenotypic Suppression	HIT	Casares F (1996)	Low	FlyBase
ABD-A	D. melanogaster	ABD-A	D. melanogaster	PCA	HIT	Hudry B (2011)	Low	FlyBase
ABD-A	D. melanogaster	ABD-B	D. melanogaster	PCA	HIT	Bischof J (2018)	Low	FlyBase
ABD-A	D. melanogaster	ABD-B	D. melanogaster	Phenotypic Suppression	BAIT	Greig S (1995)	Low	FlyBase

Green and yellow lanes indicate respectively genetic and protein interactions.
[14, 19, 20, 57-67]

Supplemental Table S3. Previously described expression vectors and reporter plasmids.

TSEII-LUC	[30]
pCMV-PBX1A	[31]
pCS2-PREP1	[32]
pCAT®-Control Vector	Promega: X65321.2
pGL4.32[luc2P/NF-kB-RE/Hygro] = Renilla standard reporter plasmid	Promega: E849A
pDest-GST	[33]
pDestFLAG = v1899 destination vector	[33]
pDestVN^{173}	[12]
pDestVC^{155}	[12]
pEnt-mHOXA1	[12]
pExpVC^{155}-mHOXA1	[12]
pExpFLAG-mHOXA1	[12]
pExpVN^{173}-hPBX1A	[12]
pExpVC^{155}-mHOXA1$^{WM\text{-}AA}$	[34]
pEnt-mHOXA1^{WMAA}	[34]
pEnt-mHOXA2	[35]
pExpFLAG-mHOXA2	[35]
pExpGST-mHOXA2	[35]
pEnt-mHOXA2^{WMAA}	[35]
pEnt-mHOXA2$^{\Delta HD}$	[36]
pEnt-mHOXA2^{HD}	[36]
pExpVN^{173}-mHOXA2	[36]
pExpVN^{173}-mHOXA2$^{\Delta HD}$	[36]
pExpVN^{173}-mHOXA2^{HD}	[36]
pExpVC^{155}-mHOXA1$^{\Delta HD}$	[28]
pEnt-mHOXA1$^{\Delta HD}$	[28]
pExpFLAG-mHOXA1$^{\Delta HD}$	[28]
pEnt-mHOXA1$^{WFQN\text{-}SVAA}$	[37]
pExpVC^{155}-mHOXA1$^{WFQN\text{-}SVAA}$	[38]
pExpFLAG-mHOXA1$^{WFQN\text{-}SVAA}$	[37]
pExpVN^{173}-mHOXA1	[38]
pExpGST-mHOXA1	[38]
pExpGST-mHOXA1$^{\Delta HD}$	[38]
pEnt-mMEIS1B	[27]

“m” and “h” stand for murine and human coding sequences respectively.

Supplemental Table S4. Primers used to generate pEnt-mHOXA5 plasmid.

Plasmids obtained	Plasmids template	Forward primer	Reverse primer
pEnt-mHOXA5	pcDNA3-mHOXA5 [39]	GGGGACAACTTTGTACAAAAAAGTTGGCATGAGCTCTTATTTTGTAAACTCATT	GGGGACAACTTTGTACAAGAAAGTTGGGCAGGGGCGGAAAGCCCCC

“m” stands for murine coding sequence.

Supplemental Table S5. Primers used to generate pEnt-mHOXA1 mutant plasmids.

Plasmids obtained	Plasmids template	Forward primer	Reverse primer
pEnt-mHOXA1^{E243A}	pEnt-mHOXA1	GGAGAAGGCGTTCCACTTCAACAAG TACC	GGTAGCGGTTGAAGTGGAATCCTT TCTCC
pEnt-mHOXA1^{E243G}	pEnt-mHOXA1	GGAGAAGGGGTTCCACTTCAACAAG TACC	GGTACTTGTTGAAGTGGAACCCCT TCTCC
pEnt-mHOXA5^{E213A}	pEnt-mHOXA5	GGAGAAAGCATTCCACTTCAACCGCT ACC	GGTAGCGGTTGAAGTGGAATGCTT TCTCC
pEnt-mHOXA5^{E213G}	pEnt-mHOXA5	GGAGAAAGGATTCCACTTCAACCGC TACC	GGTAGCGGTTGAAGTGGAATCCTT TCTCC

“m” stands for murine coding sequence.

Supplemental Table S6. Expression plasmids generated by Gateway® technology

Plasmids obtained	pEnt	pDest
pExpVC^{155}-mHOXA1^{E243A}	pEnt-mHOXA1^{E243A}	pDestVC^{155}
pExpVC^{155}-mHOXA1^{E243G}	pEnt-mHOXA1^{E243G}	pDestVC^{155}
pExpVN^{173}-mHOXA1$^{\Delta HD}$	pEnt-mHOXA1$^{\Delta HD}$	pDestVN^{173}
pExpVN^{173}-mHOXA1$^{WFQN\text{-}SVAA}$	pEnt-mHOXA1$^{WFQN\text{-}SVAA}$	pDestVN^{173}
pExpVN^{173}-mHOXA1$^{WM\text{-}AA}$	pEnt-mHOXA1$^{WM\text{-}AA}$	pDestVN^{173}
pExpVN^{173}-mHOXA1^{E243A}	pEnt-mHOXA1^{E243A}	pDestVN^{173}
pExpVN^{173}-mHOXA1^{E243G}	pEnt-mHOXA1^{E243G}	pDestVN^{173}
pExpVC^{155}-mHOXA2	pEnt-mHOXA2	pDestVC^{155}
pExpVC^{155}-mHOXA2$^{\Delta HD}$	pEnt-mHOXA2$^{\Delta HD}$	pDestVC^{155}
pExpVC^{155}-mHOXA2^{HD}	pEnt-mHOXA2^{HD}	pDestVC^{155}
pExpVC^{155}-mHOXA2^{WMAA}	pEnt-mHOXA2^{WMAA}	pDestVC^{155}
pExpVN^{173}-mHOXA2^{WMAA}	pEnt-mHOXA2^{WMAA}	pDestVN^{173}
pExpVC^{155}-mHOXA5	pEnt-mHOXA5	pDestVC^{155}
pExpVC$^{155\text{-}}$mHOXA5^{E213A}	pEnt-mHOXA5^{E213A}	pDestVC^{155}
pExpVC^{155}-mHOXA5^{E213G}	pEnt-mHOXA5^{E213G}	pDestVC^{155}
pExpVN^{173}-mHOXA5	pEnt-mHOXA5	pDestVN^{173}
pExpVN^{173}-mHOXA5^{E213A}	pEnt-mHOXA5^{E213A}	pDestVN^{173}
pExpVN$^{173\text{-}}$mHOXA5^{E213G}	pEnt-mHOXA5^{E213G}	pDestVN^{173}
pExpVN^{173}-hDLX3	pEnt-hDLX3	pDestVN^{173}
pExpVN^{173}-mMEIS1B	pEnt-MEIS1B	pDestVN^{173}
pExpFLAG-mHOXA1^{E243G}	pEnt-mHOXA1^{E243G}	pDestFLAG
pExpFLAG-mHOXA5	pEnt-mHOXA5	pDestFLAG
pExpFLAG-mHOXA5^{E213A}	pEnt-mHOXA5^{E213A}	pDestFLAG
pExpFLAG-mHOXA5^{E213G}	pEnt-mHOXA5^{E213G}	pDestFLAG
pExpGST-mHOXA1^{E243A}	pEnt-mHOXA1^{E243A}	pDestGST
pExpGST-mHOXA5	pEnt-mHOXA5	pDestGST
pExpGST-mHOXA5^{E213A}	pEnt-mHOXA5^{E213A}	pDestGST

“m” and “h” stand for murine and human coding sequences respectively.

References

1. Rezsohazy, R., et al., *Cellular and molecular insights into Hox protein action.* Development, 2015. **142**(7): p. 1212-27.
2. Alexander, T., C. Nolte, and R. Krumlauf, *Hox genes and segmentation of the hindbrain and axial skeleton.* Annu Rev Cell Dev Biol, 2009. **25**: p. 431-56.
3. Deschamps, J., *Ancestral and recently recruited global control of the Hox genes in development.* Curr Opin Genet Dev, 2007. **17**(5): p. 422-7.
4. Deschamps, J. and D. Duboule, *Embryonic timing, axial stem cells, chromatin dynamics, and the Hox clock.* Genes Dev, 2017. **31**(14): p. 1406-1416.
5. Duboule, D., *The rise and fall of Hox gene clusters.* Development, 2007. **134**(14): p. 2549-60.
6. Hubert, K.A. and D.M. Wellik, *Hox genes in development and beyond.* Development, 2023. **150**(1).
7. Barrow, J.R. and M.R. Capecchi, *Compensatory defects associated with mutations in Hoxa1 restore normal palatogenesis to Hoxa2 mutants.* Development, 1999. **126**(22): p. 5011-26.
8. Bobola, N. and S. Merabet, *Homeodomain proteins in action: similar DNA binding preferences, highly variable connectivity.* Curr Opin Genet Dev, 2017. **43**: p. 1-8.
9. Zandvakili, A. and B. Gebelein, *Mechanisms of Specificity for Hox Factor Activity.* J Dev Biol, 2016. **4**(2).
10. Bobola, N. and C.G. Sagerstrom, *TALE transcription factors: Cofactors no more.* Semin Cell Dev Biol, 2024. **152-153**: p. 76-84.
11. Merabet, S. and R.S. Mann, *To Be Specific or Not: The Critical Relationship Between Hox And TALE Proteins.* Trends Genet, 2016. **32**(6): p. 334-347.
12. Lambert, B., et al., *Protein interactions of the transcription factor Hoxa1.* BMC Dev Biol, 2012. **12**: p. 29.
13. Bondos, S.E., X.X. Tan, and K.S. Matthews, *Physical and genetic interactions link hox function with diverse transcription factors and cell signaling proteins.* Mol Cell Proteomics, 2006. **5**(5): p. 824-34.
14. Bischof, J., et al., *Generation of a versatile BiFC ORFeome library for analyzing protein-protein interactions in live Drosophila.* Elife, 2018. **7**.
15. Baeza, M., et al., *Inhibitory activities of short linear motifs underlie Hox interactome specificity in vivo.* Elife, 2015. **4**.
16. Carnesecchi, J., et al., *Multi-level and lineage-specific interactomes of the Hox transcription factor Ubx contribute to its functional specificity.* Nat Commun, 2020. **11**(1): p. 1388.
17. Kalis, A.K., et al., *Hox proteins interact to pattern neuronal subtypes in Caenorhabditis elegans males.* Genetics, 2022. **220**(4).
18. Mallen, J., et al., *Molecular Characterization of HOXA2 and HOXA3 Binding Properties.* J Dev Biol, 2021. **9**(4).
19. Papadopoulos, D.K., et al., *Dimer formation via the homeodomain is required for function and specificity of Sex combs reduced in Drosophila.* Dev Biol, 2012. **367**(1): p. 78-89.
20. Papadopoulos, D.K., et al., *Probing the kinetic landscape of Hox transcription factor-DNA binding in live cells by massively parallel Fluorescence Correlation Spectroscopy.* Mech Dev, 2015. **138 Pt 2**: p. 218-225.
21. Salomone, J., et al., *Conserved Gsx2/Ind homeodomain monomer versus homodimer DNA binding defines regulatory outcomes in flies and mice.* Genes Dev, 2021. **35**(1-2): p. 157-174.
22. Jolma, A., et al., *DNA-dependent formation of transcription factor pairs alters their binding specificity.* Nature, 2015. **527**(7578): p. 384-8.
23. Cain, B., et al., *Prediction of cooperative homeodomain DNA binding sites from high-throughput-SELEX data.* Nucleic Acids Res, 2023. **51**(12): p. 6055-6072.
24. Beachy, P.A., et al., *Cooperative binding of an Ultrabithorax homeodomain protein to nearby and distant DNA sites.* Mol Cell Biol, 1993. **13**(11): p. 6941-56.

25. Liu, Y., K.S. Matthews, and S.E. Bondos, *Internal regulatory interactions determine DNA binding specificity by a Hox transcription factor.* J Mol Biol, 2009. **390**(4): p. 760-74.
26. Galant, R., C.M. Walsh, and S.B. Carroll, *Hox repression of a target gene: extradenticle-independent, additive action through multiple monomer binding sites.* Development, 2002. **129**(13): p. 3115-26.
27. Bridoux, L., et al., *HOX paralogs selectively convert binding of ubiquitous transcription factors into tissue-specific patterns of enhancer activation.* PLoS Genet, 2020. **16**(12): p. e1009162.
28. Taminiau, A., et al., *HOXA1 binds RBCK1/HOIL-1 and TRAF2 and modulates the TNF/NF-kappaB pathway in a transcription-independent manner.* Nucleic Acids Res, 2016. **44**(15): p. 7331-49.
29. Fernandez, C.C. and L.J. Gudas, *The truncated Hoxa1 protein interacts with Hoxa1 and Pbx1 in stem cells.* J Cell Biochem, 2009. **106**(3): p. 427-43.
30. Peers, B., et al., *The pancreatic islet factor STF-1 binds cooperatively with Pbx to a regulatory element in the somatostatin promoter: importance of the FPWMK motif and of the homeodomain.* Mol Cell Biol, 1995. **15**(12): p. 7091-7.
31. Remacle, S., et al., *Changing homeodomain residues 2 and 3 of Hoxa1 alters its activity in a cell-type and enhancer dependent manner.* Nucleic Acids Res, 2002. **30**(12): p. 2663-8.
32. Goudet, G., et al., *Functional and cooperative interactions between the homeodomain PDX1, Pbx, and Prep1 factors on the somatostatin promoter.* J Biol Chem, 1999. **274**(7): p. 4067-73.
33. Rual, J.F., et al., *Towards a proteome-scale map of the human protein-protein interaction network.* Nature, 2005. **437**(7062): p. 1173-8.
34. Hudry, B., et al., *Hox proteins display a common and ancestral ability to diversify their interaction mode with the PBC class cofactors.* PLoS Biol, 2012. **10**(6): p. e1001351.
35. Bergiers, I., et al., *The homeodomain transcription factor Hoxa2 interacts with and promotes the proteasomal degradation of the E3 ubiquitin protein ligase RCHY1.* PLoS One, 2013. **8**(11): p. e80387.
36. Bridoux, L., et al., *KPC2 relocalizes HOXA2 to the cytoplasm and decreases its transcriptional activity.* Biochim Biophys Acta, 2015. **1849**(10): p. 1298-311.
37. Draime, A., et al., *The O-GlcNAc transferase OGT interacts with and post-translationally modifies the transcription factor HOXA1.* FEBS Lett, 2018. **592**(7): p. 1185-1201.
38. Draime, A., et al., *PRDM14, a putative histone methyl-transferase, interacts with and decreases the stability and activity of the HOXA1 transcription factor.* Biochim Biophys Acta Gene Regul Mech, 2018. **1861**(5): p. 534-542.
39. Coulombe, Y., et al., *Multiple promoters and alternative splicing: Hoxa5 transcriptional complexity in the mouse embryo.* PLoS One, 2010. **5**(5): p. e10600.
40. Delile, J., et al., *Single cell transcriptomics reveals spatial and temporal dynamics of gene expression in the developing mouse spinal cord.* Development, 2019. **146**(12).
41. Serrano, L., et al., *Effect of alanine versus glycine in alpha-helices on protein stability.* Nature, 1992. **356**(6368): p. 453-5.
42. Kosugi, S., et al., *Systematic identification of cell cycle-dependent yeast nucleocytoplasmic shuttling proteins by prediction of composite motifs.* Proc Natl Acad Sci U S A, 2009. **106**(25): p. 10171-6.
43. Nguyen Ba, A.N., et al., *NLStradamus: a simple Hidden Markov Model for nuclear localization signal prediction.* BMC Bioinformatics, 2009. **10**: p. 202.
44. Robinson, G.W. and K.A. Mahon, *Differential and overlapping expression domains of Dlx-2 and Dlx-3 suggest distinct roles for Distal-less homeobox genes in craniofacial development.* Mech Dev, 1994. **48**(3): p. 199-215.
45. Depew, M.J., et al., *Reassessing the Dlx code: the genetic regulation of branchial arch skeletal pattern and development.* J Anat, 2005. **207**(5): p. 501-61.
46. Remacle, S., et al., *Loss of function but no gain of function caused by amino acid substitutions in the hexapeptide of Hoxa1 in vivo.* Mol Cell Biol, 2004. **24**(19): p. 8567-75.

47. Phelan, M.L. and M.S. Featherstone, *Distinct HOX N-terminal arm residues are responsible for specificity of DNA recognition by HOX monomers and HOX.PBX heterodimers.* J Biol Chem, 1997. **272**(13): p. 8635-43.
48. Bridoux, L., F. Gofflot, and R. Rezsohazy, *HOX Protein Activity Regulation by Cellular Localization.* J Dev Biol, 2021. **9**(4).
49. Deneyer, N., et al., *HOXA2 activity regulation by cytoplasmic relocation, protein stabilization and post-translational modification.* Biochim Biophys Acta Gene Regul Mech, 2019. **1862**(9): p. 194404.
50. Luck, K., et al., *A reference map of the human binary protein interactome.* Nature, 2020. **580**(7803): p. 402-408.
51. Ravasi, T., et al., *An atlas of combinatorial transcriptional regulation in mouse and man.* Cell, 2010. **140**(5): p. 744-52.
52. Rolland, T., et al., *A proteome-scale map of the human interactome network.* Cell, 2014. **159**(5): p. 1212-1226.
53. Yu, H., et al., *Next-generation sequencing to generate interactome datasets.* Nat Methods, 2011. **8**(6): p. 478-80.
54. Huttlin, E.L., et al., *Dual proteome-scale networks reveal cell-specific remodeling of the human interactome.* Cell, 2021. **184**(11): p. 3022-3040 e28.
55. Pourhaghighi, R., et al., *BraInMap Elucidates the Macromolecular Connectivity Landscape of Mammalian Brain.* Cell Syst, 2020. **11**(2): p. 208.
56. Wang, X.W., et al., *Assessment of community efforts to advance network-based prediction of protein-protein interactions.* Nat Commun, 2023. **14**(1): p. 1582.
57. Percival-Smith, A., W.A. Teft, and J.L. Barta, *Tarsus determination in Drosophila melanogaster.* Genome, 2005. **48**(4): p. 712-21.
58. Hirth, F., et al., *Functional equivalence of Hox gene products in the specification of the tritocerebrum during embryonic brain development of Drosophila.* Development, 2001. **128**(23): p. 4781-8.
59. Boube, M., et al., *Drosophila homologs of transcriptional mediator complex subunits are required for adult cell and segment identity specification.* Genes Dev, 2000. **14**(22): p. 2906-17.
60. Percival-Smith, A. and J.A. Bondy, *Analysis of murine HOXA-2 activity in Drosophila melanogaster.* Dev Genet, 1999. **24**(3-4): p. 336-44.
61. Greig, S. and M. Akam, *The role of homeotic genes in the specification of the Drosophila gonad.* Curr Biol, 1995. **5**(9): p. 1057-62.
62. Kennison, J.A. and J.W. Tamkun, *Dosage-dependent modifiers of polycomb and antennapedia mutations in Drosophila.* Proc Natl Acad Sci U S A, 1988. **85**(21): p. 8136-40.
63. Hannah-Alava, A., *Interaction of Non-Allelic Loci in Expression of the Extra-Sexcomb Phenotype in Drosophila Melanogaster.* Z Vererbungsl, 1964. **95**: p. 1-9.
64. Li, X. and W. McGinnis, *Activity regulation of Hox proteins, a mechanism for altering functional specificity in development and evolution.* Proc Natl Acad Sci U S A, 1999. **96**(12): p. 6802-7.
65. Hudry, B., et al., *Visualization of protein interactions in living Drosophila embryos by the bimolecular fluorescence complementation assay.* BMC Biol, 2011. **9**: p. 5.
66. Casares, F., M. Calleja, and E. Sanchez-Herrero, *Functional similarity in appendage specification by the Ultrabithorax and abdominal-A Drosophila HOX genes.* EMBO J, 1996. **15**(15): p. 3934-42.
67. de Navas, L., et al., *A simple and efficient method to identify replacements of P-lacZ by P-Gal4 lines allows obtaining Gal4 insertions in the bithorax complex of Drosophila.* Mech Dev, 2006. **123**(11): p. 860-7.
